# Multiple crosstalk between TOR and the cell integrity MAPK signaling pathway in fission yeast

**DOI:** 10.1038/srep37515

**Published:** 2016-11-23

**Authors:** Marisa Madrid, Beatriz Vázquez-Marín, Alejandro Franco, Teresa Soto, Jero Vicente-Soler, Mariano Gacto, José Cansado

**Affiliations:** 1 Yeast Physiology Group, Department of Genetics and Microbiology, Facultad de Biología, Universidad de Murcia, 30071 Murcia, Spain

## Abstract

In eukaryotic cells, the highly conserved Target of Rapamycin (TOR) and the Mitogen Activated Protein Kinase (MAPK) signaling pathways elicit adaptive responses to extra- and intracellular conditions by regulating essential cellular functions. However, the nature of the functional relationships between both pathways is not fully understood. In the fission yeast *Schizosaccharomyces pombe* the cell integrity MAPK pathway (CIP) regulates morphogenesis, cell wall structure and ionic homeostasis. We show that the Rab GTPase Ryh1, a TORC2 complex activator, cross-activates the CIP and its core member, the MAPK Pmk1, by two distinct mechanisms. The first one involves TORC2 and its downstream effector, Akt ortholog Gad8, which together with TORC1 target Psk1 increase protein levels of the PKC ortholog Pck2 during cell wall stress or glucose starvation. Also, Ryh1 activates Pmk1 in a TORC2-independent fashion by prompting plasma membrane trafficking and stabilization of upstream activators of the MAPK cascade, including PDK ortholog Ksg1 or Rho1 GEF Rgf1. Besides, stress-activated Pmk1 cross-inhibits Ryh1 signaling by decreasing the GTPase activation cycle, and this ensures cell growth during alterations in phosphoinositide metabolism. Our results reveal a highly intricate cross-regulatory relationship between both pathways that warrants adequate cell adaptation and survival in response to environmental changes.

The Target of Rapamycin (TOR) and the Mitogen Activated Protein Kinase (MAPK) cascades are signaling pathways conserved in eukaryotic organisms that control adaptive responses to extra- and intracellular conditions^1^,^2^. Their misregulation may cause diabetes, cancer, aging, autoimmune diseases, or developmental abnormalities^1^,^2^,^3^,^4^. TOR is found as two different multiprotein complexes, TORC1 and TORC2^5^. TORC1 controls essential cellular functions, including transcription, protein and lipid synthesis, ribosome biogenesis, nutrient transport and autophagy, whereas TORC2 regulates actin cytoskeleton polarization, cell cycle progression, endocytosis, calcineurin activity, sphingolipid biosynthesis, and membrane homeostasis^3^,^5^,^6^. Some of the above TORC2-related functions (actin cytoskeleton organization, sphingolipid biosynthesis, …), have been described in budding yeast *S. cerevisiae* and might not be conserved in TORC2 complexes in other eukaryotes.

Multiple MAPK pathways are present along the eukaryotic lineage. They regulate gene expression, mRNA stabilization and translation, cell cycle progression, proliferation, differentiation, and cell survival and adaptation in response to environmental changes^2^. Remarkably, some of these processes are also regulated by TOR complexes, suggesting that TOR and MAPK signaling pathways may crosstalk. Indeed, in mammalian cells the Ras-ERK (MAPK) and PI3K-mTORC1 pathways up- or down-regulate each other^7^. MAPK p38 isoforms also affect mTORC1 activation^5^. In *Saccharomyces cerevisiae* and *Candida albicans* the activity of the respective cell integrity MAPKs Slt2/Mpk1 and Mkc1 (ERK orthologs) is altered in response to TORC1 inhibition^8^,^9^. In contrast, much less is known about the relationship between MAPKs and the TORC2 complex.

Fission yeast *Schizosaccharomyces pombe* is an excellent model to study TOR and MAPK signaling^10^,^11^. Fission yeast TORC1 includes the catalytic subunit Tor2 (Tor1 in budding yeast)^10^. Similar to mammalian cells, its activity is regulated by Rheb GTPase ortholog Rhb1^12^. TORC1 is essential for vegetative growth, and becomes activated by nutrients to enhance ribosome biogenesis and protein synthesis, while controlling negatively sexual differentiation^13^,^14^,^15^. Among TORC1 targets is the AGC kinase Psk1, which is the major S6 kinase in this organism and responds to nutrient availability^16^. TORC2, which includes the catalytic subunit Tor1 (Tor2 in budding yeast), is nonessential, but regulates several aspects of cell cycle progression and cell survival upon DNA damage, and after stress conditions^17^,^18^. The AGC-kinase Gad8 (Akt ortholog) is the main target for TORC2, and most defects in Tor1-less mutants are phenocopied by *gad8∆* cells^19^,^20^. Gad8 biological functions are dependent upon activation loop phosphorylation at T387 by Ksg1 (PDK1), together with phosphorylation of both S527 (turn motif) and S546 (hydrophobic motif) by Tor1^20^. The small Rab GTPase Ryh1, ortholog to human Rab6, is the main TORC2 activator in fission yeast and the only known activator of a TORC2 complex within this class of proteins^5^,^21^. GTP-bound Ryh1 associates with TORC2 to induce phosphorylation and activation of Gad8 during growth, and its GTPase activity is strongly reduced under glucose starvation^22^. Ryh1 localizes mainly to the Golgi apparatus, and also regulates membrane trafficking from endosomes to Golgi, ER, and plasma membrane^23^,^24^,^25^.

The cell integrity MAPK pathway (CIP) regulates in *S. pombe* cell wall construction and maintenance during stress, cytokinesis, morphogenesis, mRNA stabilization and ionic homeostasis^11^,^26^. Its core member, ERK ortholog MAPK Pmk1, is activated by adverse conditions such as hyperosmotic stress, cell wall damage or glucose withdrawal^11^,^27^,^28^. Rho GTPases Rho1 and Rho2 are the main positive regulators of the CIP through Pck2, one of the two orthologs of protein kinase C (PKC) in this organism^28^. Similar to Gad8 and Psk1, Pck2 activation requires activation loop phosphorylation by Ksg1 (PDK1), and undergoes subsequent autophosphorylation of the turn motif^29^. These events, together with binding to Rho1 and/or Rho2, stabilize and render Pck2 catalytically competent to modulate cell integrity through activation of Pmk1^29^. We recently found that increased *de novo* Pck2 synthesis is essential to trigger Pmk1 activation in response to cell wall damage or glucose starvation, and that TORC2 is involved in such control^27^,^29^. Also, Pmk1 might negatively regulate the TORC2-Gad8 signaling^30^, suggesting that the CIP and TORC2 pathways extensively crosstalk *in vivo*, although the mechanisms involved are currently unknown.

In this work we show that TORC2-Gad8 and Psk1 increase Pck2 levels that contributes to Pmk1 activation during glucose starvation and cell wall stress. Ryh1 also induces MAPK activation in a TORC2-Gad8 independent fashion by eliciting plasma membrane targeting and/or stabilization of several upstream activators of the CIP. Notably, PI kinase Its3 and PI(4,5)P2 promote Ryh1-TORC2 signaling and activation of the CIP, likely acting in this latter case as a Ryh1 effector. Finally, activated Pmk1 cross-inhibits TORC2-Gad8 signaling by decreasing Ryh1 activity. Our observations highlight the multifaceted nature of the crosstalk mechanisms elicited by these two major conserved signaling pathways to ensure adequate cell adaptation and survival in response to environmental changes.

## Results

### TORC2 target Gad8 regulates Pck2 levels and MAPK activation during cell wall stress and glucose deprivation

**Figure 1:**
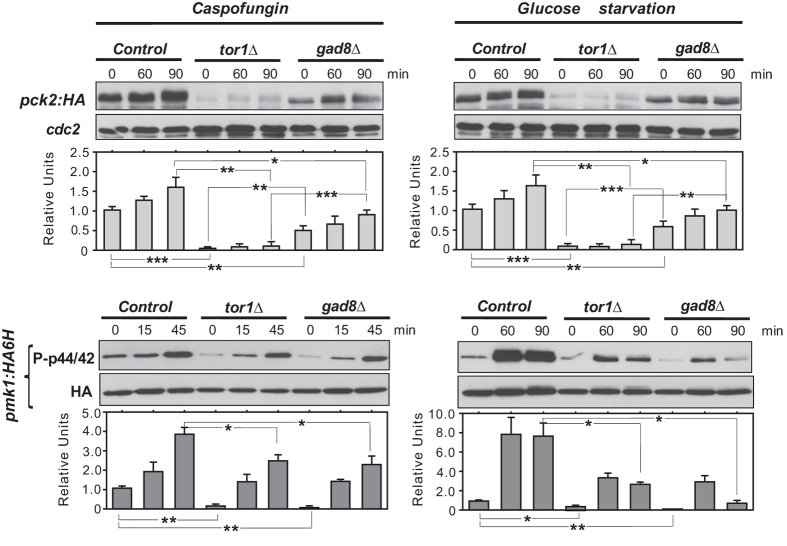
Gad8 regulates Pck2 levels and Pmk1 activation during growth, cell wall stress and glucose deprivation. *Upper panels*. Growing cultures of strains MM913 (Pck2-HA; control), MM1205 (*tor1Δ* Pck2-HA), and BV11 (*gad8Δ* Pck2-HA) expressing genomic Pmk1-HA6H fusions were treated with 1 μg/ml Caspofungin (left panel), or starved for glucose (right panel). Cell extracts were resolved by SDS-PAGE and Pck2 levels detected with anti-HA antibodies. Anti-Cdc2 was used as loading control. *Lower panels*. Pmk1-HA6H fusion was purified by affinity chromatography, and activated/total Pmk1 detected with anti-phospho-p44/42 and anti-HA antibodies, respectively. *P < 0.05; **P < 0.005; ***P < 0.001.

The fission yeast TORC2 complex does not participate in catalytic activation of Pck2. Nevertheless, this complex contributes to *de novo* Pck2 synthesis, which is essential to activate the CIP in response to cell wall damage or glucose exhaustion, but not to other stimuli like saline stress^29^. To identify the main components of this novel regulatory mechanism, we analyzed Pck2 levels and Pmk1 activation in a series of mutants lacking upstream regulators or downstream effectors of the TORC2 signaling cascade. As expected, Pck2 levels were strongly reduced in *tor1∆* cells during growth and under cell wall stress (Caspofungin) or glucose deprivation as compared to control cells, resulting in a delayed or lowered Pmk1 activation, respectively (Fig. 1). Notably, similar to *tor1∆* cells, Pck2 levels decreased in growing and stressed cells lacking the AGC-kinase family member Gad8, the major target for TORC2 (Fig. 1)^20^. However, defective Pck2 levels in *gad8∆* cells were less severe than in the *tor1∆* mutant, although showed a similar defect in Pmk1 activation (Fig. 1). Hence, while both basal and increased Pck2 levels in response to the above stimuli is mostly dependent on the Tor1-Gad8 branch, Gad8 does not appear to be the sole Tor1-target controlling this response.

### TORC1-Psk1 cooperates with TORC2-Gad8 to promote stress-induced Pck2 levels and Pmk1 activation

**Figure 2:**
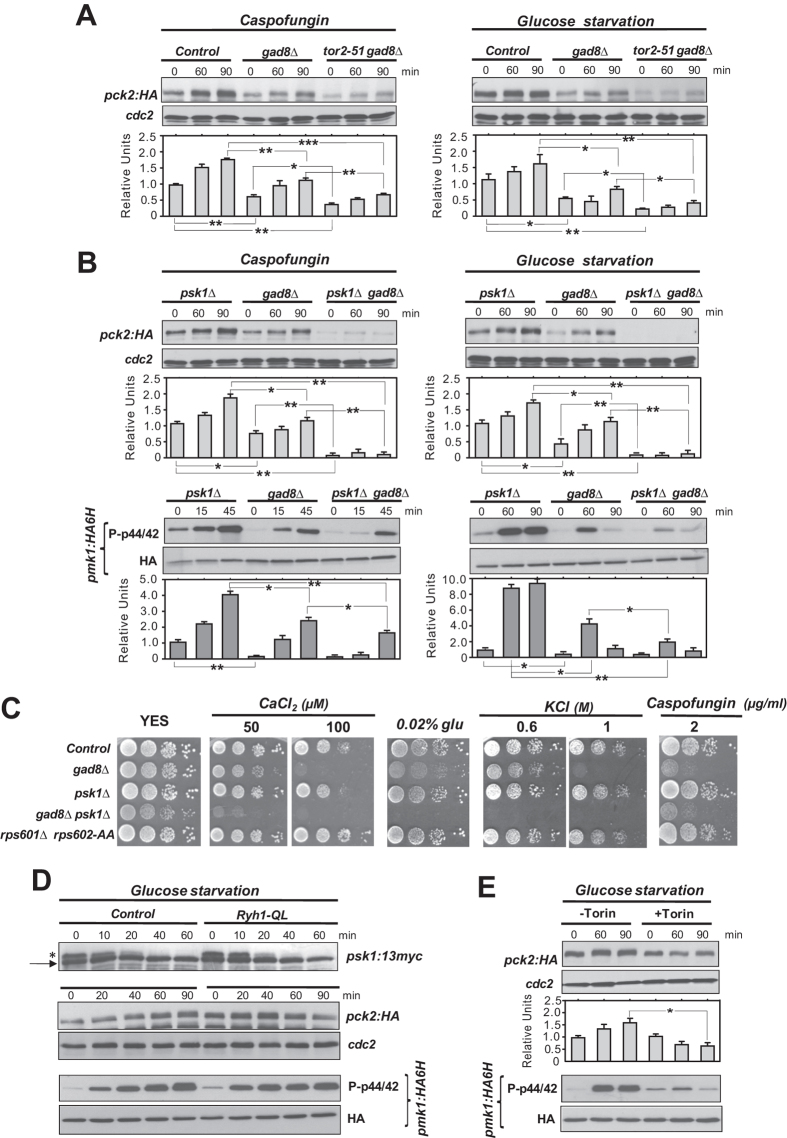
TORC1-Psk1 participates with TORC2-Gad8 in increasing Pck2 levels and Pmk1 activation during growth and stress. (**A**) Cultures of strains BV8 (Pck2-HA; control), BV11 (*gad8Δ* Pck2-HA), and BV369 (*tor2-51 gad8Δ* Pck2-HA) were grown at 25 °C, incubated at 36 °C for one hour, and treated with 1 μg/ml Caspofungin (left panel), or starved for glucose (right panel). Cell extracts were resolved by SDS-PAGE and Pck2 levels detected after incubation with anti-HA antibodies. Anti-Cdc2 was used as a loading control. (**B**) *Upper panels*. Growing cultures of strains BV13 (*psk1Δ* Pck2-HA), BV11 (*gad8Δ* Pck2-HA), and BV14 (*psk1Δ gad8Δ* Pck2-HA) expressing genomic Pmk1-HA6H fusions were treated with Caspofungin (left panels), or starved for glucose (right panels), and Pck2 levels were detected as described above. *Lower panels*. Pmk1-HA6H fusion was purified by affinity chromatography, and activated/total Pmk1 detected with anti-phospho-p44/42 and anti-HA antibodies, respectively. *P < 0.05; **P < 0.005. (**C**) Serial dilutions of suspensions of strain BV78 (*rps601Δ rps602-AA* Pck2-HA) and of those described in (A) and (B), were spotted on YES plates supplemented with different concentrations of CaCl_2_, KCl, Caspofungin, or glucose, and incubated for 3 or 5 days (low glucose plates) at 28 °C. (**D**) Strains AN0179 (Psk1-13myc) and BV90 (Ryh1-QL Psk1-13myc) expressing genomic Pmk1-HA6H fusions were grown in YES medium and starved for glucose. The Psk1-13myc fusion was detected after incubation with anti-myc antibodies. Pck2 levels were detected as described above. Activated/total Pmk1 detected with anti-phospho-p44/42 and anti-HA antibodies, respectively. *Arrow*, unphosphorylated Psk1; *asterisk*, TORC1-phosphorylated Psk1. (**E**) A growing culture of control strain BV8 (Pck2-HA) expressing a genomic Pmk1-HA6H fusion was incubated with 50 μM Torin for 60 min or remained untreated (DMSO). These cells were then starved for glucose for the indicated times. Pck2 was detected as described above. Activated/total Pmk1 detected with anti-phospho-p44/42 and anti-HA antibodies, respectively. *P < 0.05 in Torin–treated cells as compared to the untreated culture.

A mutant strain expressing the hypomorphic thermo-sensitive allele *tor2-51*^14^ does not show defective Pck2 levels during vegetative growth, cell wall stress or glucose deprivation (Suppl. Figure S1). However, introduction of this mutation in a *gad8∆* background (*tor2-51 gad8∆* cells) elicited a further and significant decrease in Pck2 levels under each stress condition (Fig. 2A). Deletion of the S6 kinase ortholog Psk1, which becomes phosphorylated by TORC1 in the presence of nitrogen and carbon sources^16^, did not modify Pck2 levels during the above conditions (Fig. 2B). Strikingly, the defective Pck2 levels in growing or stressed *gad8∆* cells were strongly reduced in a *gad8∆ psk1∆* double mutant, and this prompted an additive drop in Pmk1 activation, particularly in the absence of glucose (Fig. 2B). When compared to control cells, *gad8∆* cells displayed a clear growth defect in response to elevated calcium concentrations (CaCl_2_), osmotic saline stress (KCl), cell wall damage (Caspofungin), or upon limited glucose concentrations (Fig. 2C). On the contrary, *psk1∆* cells did not show stress sensitivity (Fig. 2C). Remarkably, although slow growing, the stress sensitivity of the *gad8∆ psk1∆* double mutant was in many instances aggravated in comparison to that of *gad8∆* cells (Fig. 2C). Taken together, these results support that Psk1 cooperates with Tor1-Gad8 (TORC2) in the control of adaptation to stress, Pck2 levels, and Pmk1 activation, although its role appears to be less prominent than that of the Tor1-Gad8 branch.

The observation that downregulation of Pck2 levels in *gad8∆* cells is less pronounced than in *tor1∆* or *gad8∆ psk1∆* mutants (Figs 1 and 2B), suggests that TORC2 might regulate Psk1 activity to some extent. TORC1 phosphorylates Psk1 *in vivo* at T415 within its hydrophobic motif in a nutrient-dependent manner, and this event can be detected as a mobility band-shift by SDS-PAGE (Fig. 2D). Psk1 phosphorylation was totally absent in the *tor2-51* mutant and in control cells treated with the ATP-analogue Torin-1, that targets the kinase domain of mTOR and inhibits the *in vivo* activity of both TORC1 and TORC2 complexes in fission yeast^31^ (Suppl. Figure S1). Moreover, the rate of Psk1 dephosphorylation in glucose-starved cells was identical in control, *tor1∆* cells, and in a mutant expressing a constitutively active (GTP-locked) version of the Ryh1 GTPase (Ryh1-*Q70L*), which in fission yeast activates the TORC2-Gad8 branch in response to glucose availability^22^ (Fig. 2D; Suppl. Figure S1). In this mutant, basal Pck2 levels and Pmk1 activity were slightly higher than in control cells (Fig. 2D), thus confirming the positive role of Ryh1-TORC2 in CIP signalling. Nevertheless, total Pck2 and phosphorylated Pmk1 raised similarly in both strains during glucose starvation (Fig. 2D). Finally, contrary to untreated cells, pretreatment of wild type cells with 50 μM Torin1 for 1 hour resulted in decreased Pck2 levels in glucose-starved and Caspofungin-treated cells, and this prompted a strong inhibition of Pmk1 activation (Fig. 2E; Suppl. Figure S1). Hence, TORC1 and TORC2 specifically target Psk1 and Gad8, respectively, to regulate Pck2 levels and response to stress.

### Gad8 and Psk1 regulate Pck2 levels during stress independently of Rps6 phosphorylation

**Figure 3:**
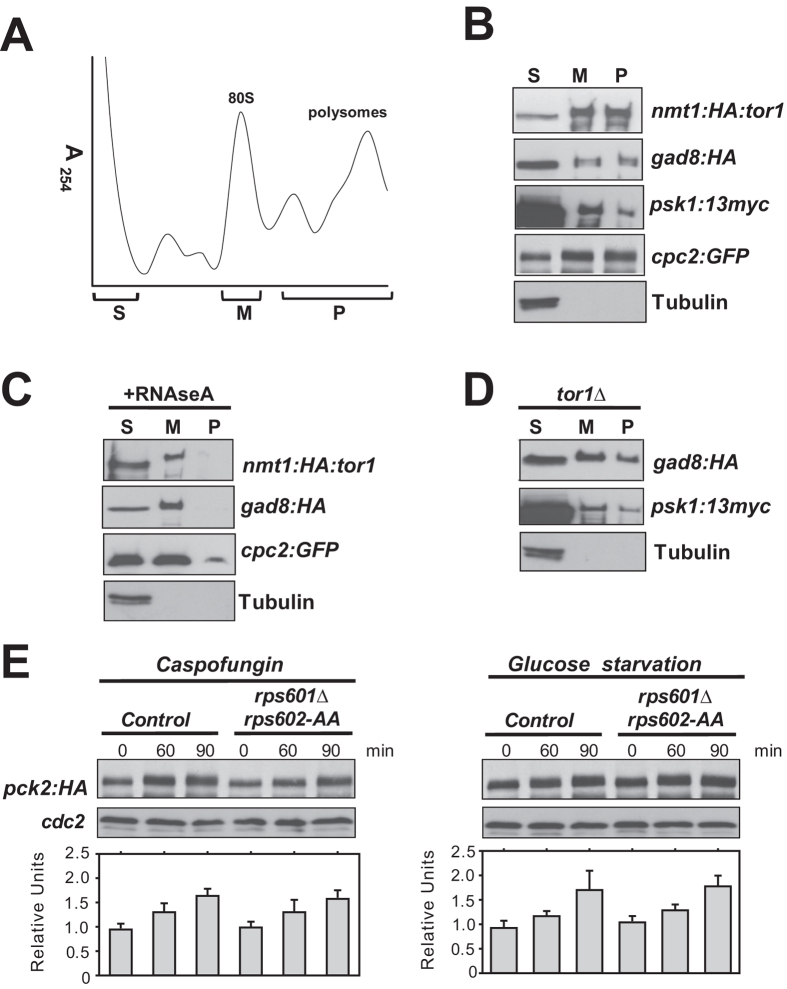
Tor1, Gad8, and Psk1 associate to translating ribosomes, and regulate Pck2 levels during stress independently of Rps6 phosphorylation. (**A**) Absorbance profile at 254 nm of a representative polysome sucrose gradient. Soluble (S), monosomal (M), and polysomal (P) fractions are indicated. (**B**) Ribosomes from cycloheximide-treated cultures of strains BA192 (*nmt1*-HA-Tor1), JW960 (Gad8-HA), AN0179 (Psk1-13myc), and AN071 (Cpc2-GFP; 40 S ribosomal protein), were purified by sedimentation through sucrose gradients. Identical volumes of representative monosomal and polysomal fractions were analyzed by Western blot with either anti-HA, anti-Myc, or anti-GFP antibodies. Anti-tubulin was used as a non-ribosomal negative control. (**C**) Cell extracts from strains described in (**B**) were incubated with 0.5 mg/ml RNAse A for 20 min before sucrose sedimentation, and monosomal and polysomal fractions analyzed as above. (**D**) Western blot analysis of monosomal and polysomal ribosomal fractions from cycloheximide-treated cultures of strains BV397 (*tor1Δ* Gad8-HA), and BV84 (*tor1Δ* Psk1-13myc). (**E**) Growing cultures of strains BV8 (Pck2-HA; control) and BV78 (*rps601Δ rps602-AA* Pck2-HA) were treated with 1 μg/ml Caspofungin (left panel), or shifted to the same medium without glucose (right panel). Pck2 levels were detected after incubation with anti-HA antibodies. Anti-Cdc2 was used as loading control. *P < 0.05 in mutant strain as compared to the control.

In mammalian cells mTORC2 association with ribosomes is required for its activation and downstream signaling^32^. We performed sucrose gradient fractionation to investigate the putative *in vivo* interaction of Tor1, Gad8, and/or Psk1 with ribosomes. As previously described, a GFP-fused version of Cpc2, the fission yeast ortholog of mammalian RACK1 and a structural component of the 40 S ribosomal subunit^33^, distributed throughout the gradient within 40 S, monosome, and polysome fractions. On the contrary, tubulin (non-ribosomal protein; negative control), was detected exclusively in the soluble fraction (Fig. 3A,B). Importantly, HA-Tor1, Gad8-HA, and Psk1-13myc fusions also co-sedimented in monosomal and polysomal fractions similar to Cpc2 (Fig. 3B). In these assays a noticeable amount of Tor1 appeared to be constitutively associated to monosomes and/or polysomes, while the ribosome-bound pool of Gad8 and Psk1 was clearly lower than in the soluble fraction (Fig. 3B). When cell extracts were treated before sedimentation with RNAse A, which dissociates polyribosomes to free ribosomes, Cpc2, Tor1, Gad8, and Psk1 were mostly found in the monosome fractions (Fig. 3C), thus confirming its association to this specific organelle. Notably, the association of Gad8 and Psk1 with ribosomes was still maintained in *tor1∆* cells (Fig. 3D). Therefore, in fission yeast TORC2 and its target Gad8 associate with both translating and non-translating ribosomes, and Gad8-ribosome interaction is independent of the presence of TORC2.

The essential and evolutionary conserved ribosomal protein Rps6 is involved in many physiological roles including the control of cell size, cell proliferation, and global protein synthesis^34^. In fission yeast, Rps6 is encoded by two genes, *rps601*+ and *rps602*+, and results phosphorylated *in vivo* at the conserved serine residues at positions 235 and 236 by both Psk1 and Gad8 in response to nitrogen source and glucose^13^,^35^. However, *rps601*Δ *rps602-AA* mutant cells lacking Ser-235 and Ser-236 did not show growth sensitivity under multiple stress conditions in contrast to *gad8∆ psk1∆* cells (Fig. 2C). Besides, Pck2 levels were not significantly affected during growth with Caspofungin or upon glucose withdrawal (Fig. 3E). Hence, Gad8-Psk1 regulation of Pck2 levels and Pmk1 activation during stress does not operate through Rps6 phosphorylation.

### Ryh1 GTPase modulates cell integrity signaling through TORC2-dependent and -independent mechanisms

**Figure 4:**
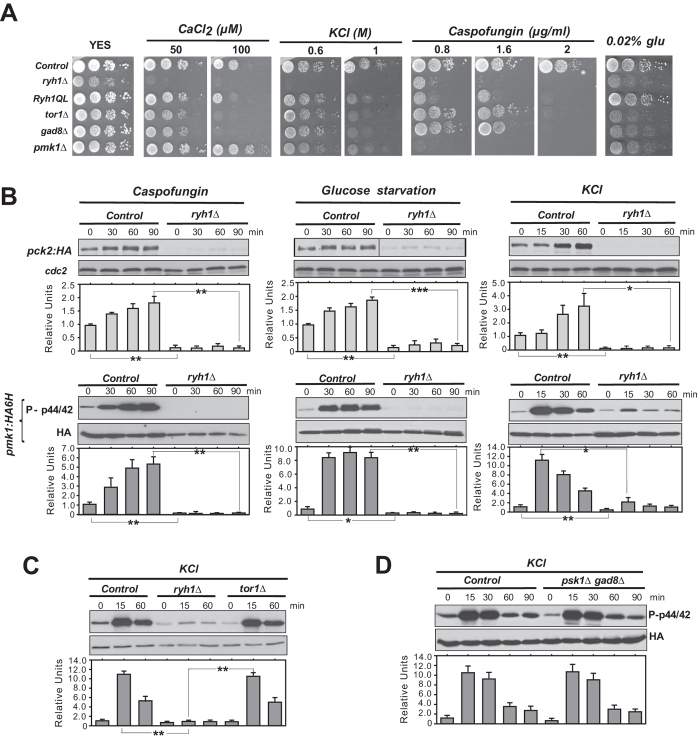
Rab-family GTPase Ryh1 regulates the cell integrity pathway in a TORC2-dependent and -independent fashion. (**A**) Serial dilutions of suspensions of strains MM913 (Pck2-HA; control), BV38 (*ryh1Δ* Pck2-HA), BV56 (*Ryh1-QL* Pck2-HA), MM1205 (*tor1Δ* Pck2-HA), BV11 (*gad8Δ* Pck2-HA), and MM1200 (*pmk1Δ* Pck2-HA), were spotted on YES plates supplemented with different concentrations of CaCl_2_, KCl, Caspofungin, or glucose, and incubated for 3 or 5 days at 28 °C. (**B**) *Upper panels*. Growing cultures of strains BV8 (Pck2-HA; control), and BV38 (*ryh1Δ* Pck2-HA) expressing genomic Pmk1-HA6H fusions were treated with 1 μg/ml Caspofungin (left panel), starved for glucose (middle panel), or treated with 0.6 M KCl (right panel). Cell extracts were resolved by SDS-PAGE and Pck2 levels detected after incubation with anti-HA antibodies. Anti-Cdc2 was used as a loading control. *Lower panels*. Purification and detection of activated/total Pmk1 was performed as described above. *P < 0.05; **P < 0.005; ***P < 0.001. (**C**) Growing cultures of strains BV8 (Pck2-HA; control), BV38 (*ryh1Δ* Pck2-HA) and MM1205 (*tor1Δ* Pck2-HA) expressing genomic Pmk1-HA6H fusions were treated with 0.6 M KCl for the indicated times. Activated/total Pmk1 were detected with anti-phospho-p44/42 and anti-HA antibodies, respectively. **P < 0.005 (**D**) Growing cultures of strains BV8 (Pck2-HA; control) and BV14 (*psk1Δ gad8Δ* Pck2-HA) expressing genomic Pmk1-HA6H fusions were treated with 0.6 M KCl. Purification and detection of activated/total Pmk1 was performed as described above.

In its GTP-bound form, Rab-family GTPase Ryh1 associates *in vivo* and activates TORC2 to promote phosphorylation and activation of Gad8^21^. As compared to control cells, *ryh1∆* cells displayed strong growth sensitivity to CaCl_2_, KCl, Caspofungin, or low glucose concentrations (Fig. 4A). Many of these phenotypes are shared by *tor1∆* or *gad8∆* mutants, but the overall sensitivity to stress was more pronounced in *ryh1∆* cells (Fig. 4A), suggesting that Ryh1 signaling in response to environmental changes might be elicited through additional mechanisms different to those mediated by TORC2-Gad8. Similar to *tor1∆* cells, Pck2 levels were strongly abrogated in *ryh1∆* cells during growth and in response to cell wall stress or upon glucose removal (Fig. 4B), and the drop in Pmk1 activation was even stronger than in *tor1∆* cells (Fig. 1). Remarkably, Pmk1 activation was also very low in *ryh1∆* cells treated with KCl (Fig. 4B). This finding was unexpected, since Pmk1 became strongly activated by this stimulus in *tor1∆* cells (Fig. 4C). Moreover, Pmk1 activation in salt stressed *gad8∆ psk1∆* cells was similar to that of control cells (Fig. 4D). Thus, Ryh1 positively regulates the cell integrity pathway through additional, TORC2-independent mechanisms. Such control seems specific for the cell integrity pathway, since both the magnitude and kinetics of activation of Sty1, the core member of the stress activated MAPK pathway (SAPK) in fission yeast^11^, was identical in control and *ryh1∆* cells under salt stress (Suppl. Figure S2). Human Rab6 GTPase can stimulate TORC2-Gad8 signaling *S. pombe*^21^. Indeed, *ryh1∆* cells expressing a cDNA encoding human Rab6 under the control of the *ryh1 *+* *5´UTR effectively suppressed the growth sensitivity of the parental mutant strain in the presence of CaCl_2_, KCl, Caspofungin, or low glucose concentrations (Suppl. Figure S3). This was accompanied by a moderate increase in the Pck2 levels during glucose starvation, and in Pmk1 activation by saline stress (Suppl. Figure S3). Hence, this novel regulatory role for Ryh1 might be evolutionary conserved.

### Ryh1 promotes proper plasma membrane tethering and/or processing of upstream activators of the CIP independently of TORC2 signaling

**Figure 5:**
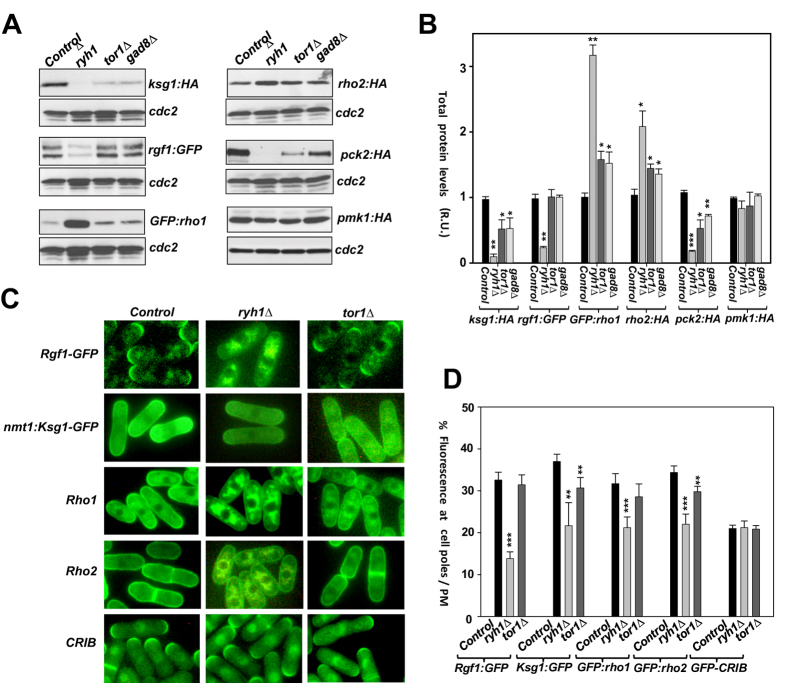
Ryh1 promotes proper plasma membrane localization and/or processing of upstream activators of the cell integrity pathway. (**A**) Total extracts from growing cultures of control, *ryh1Δ, tor1Δ*, and *gad8Δ* strains expressing Ksg1-HA, Rgf1-GFP, GFP-Rho1, Rho2-HA, Pck2-HA, and Pmk1-HA fusions were resolved by SDS-PAGE and the levels of the respective fusions detected by incubation with anti-HA or anti-GFP antibodies. Anti-Cdc2 was used as a loading control. (**B**) Quantification of Western blot experiments shown in (A). *P < 0.05; **P < 0.005; ***P < 0.001. (**C**) Images by fluorescence microscopy of growing control, *ryh1Δ,* and *tor1Δ* cells expressing either Rgf1-GFP, nmt1:Ksg1-GFP, GFP-Rho1, GFP-Rho2, or GFP-CRB fusions. (**D**) The percentage at the plasma membrane/cell tips of the GFP fusions described in (C) with respect to total cell fluorescence was determined. N > 15 cells; **P < 0.005; ***P < 0.001.

Ryh1 and orthologs like mammalian Rab6 or budding yeast Ypt6 are major regulators of Golgi membrane trafficking in eukaryotic cells^23^. The main upstream activators of the CIP, PDK ortholog Ksg1, Rho1 GEF Rgf1, and GTPases Rho1 and Rho2, activate Pmk1 via Pck2 during growth and in response to stress^27^,^36^,^37^. Importantly, they are mostly targeted to the plasma membrane and/or cell poles^36^,^37^. We therefore hypothesized that Ryh1 might regulate the activity of the CIP independently of TORC2 signaling by promoting an adequate processing and/or trafficking of one or several upstream regulators of this signaling cascade. Indeed, protein levels of Ksg1were very low in *ryh1∆* cells as compared to control cells (Fig. 5A,B). *tor1∆* and *gad8∆* cells also showed some decrease in Ksg1 levels, but this defect was not as evident as in the *ryh1∆* mutant (Fig. 5A,B). Protein levels of Rgf1were also strongly down-regulated in *ryh1∆* cells, but remained unchanged in *tor1∆* and *gad8∆* cells (Fig. 5A,B).

Rgf1 targeting to the cell tips was clearly reduced in *ryh1∆* cells as compared to control cells, and remained unaffected in the *tor1∆* mutant (Fig. 5C,D). Similarly, the relative fluorescence at the plasma membrane of Ksg1-GFP, GFP-Rho1, and GFP-Rho2 fusions was lower in *ryh1∆* cells than in control or *tor1∆* cells (Fig. 5C,D). GFP-Rho1 and GFP-Rho2 accumulated in *ryh1∆* cells in structures resembling internal membranes (Fig. 5C). Remarkably, protein levels of both GTPases increased in *ryh1∆* cells *versus* control cells, and this effect was less evident in the *tor1∆* and *gad8∆* mutants (Fig. 5A,B). Absence of prenylation decreases Rho2 targeting to the plasma membrane while increasing total GTPase levels due to a defective turnover at this location^37^. Thus, reduced prenylation might explain the increased protein levels of both GTPases observed in cells lacking Ryh1. Nonetheless, Ryh1 regulation of Rho1 and Rho2 plasma membrane trafficking seems specific for both Rho GTPase members as localization at cell tips of active GTP-bound Cdc42 (GFP-CRIB)^38^ was not differentially altered in control *versus ryh1∆* or *tor1∆* cells (Fig. 5C). Finally, Pck2 protein levels, were further reduced in *ryh1∆* cells respect to either *tor1∆* or *gad8∆* mutants, while total levels of Pmk1 remained very similar in control, *ryh1∆*, *tor1∆,* or *gad8∆* cells (Fig. 5A,B). The strongest decrease in Pck2 levels observed in *ryh1∆* cells may then emerge as a direct consequence of Ksg1, Rgf1, Rho1, and Rho2 mislocalization, as they regulate phosphorylation and stabilization of Pck2 *in vivo*^27^,^36^,^37^. Taken together, the above observations suggest that Ryh1 may activate cell integrity signaling independently of TORC2 by eliciting adequate plasma membrane trafficking and/or processing of specific upstream activators (Ksg1, Rgf1, Rho1, Rho2) of this signaling cascade.

### MAPK Pmk1 acts upstream Ryh1 to inhibit TORC2 signaling in response to stress

**Figure 6:**
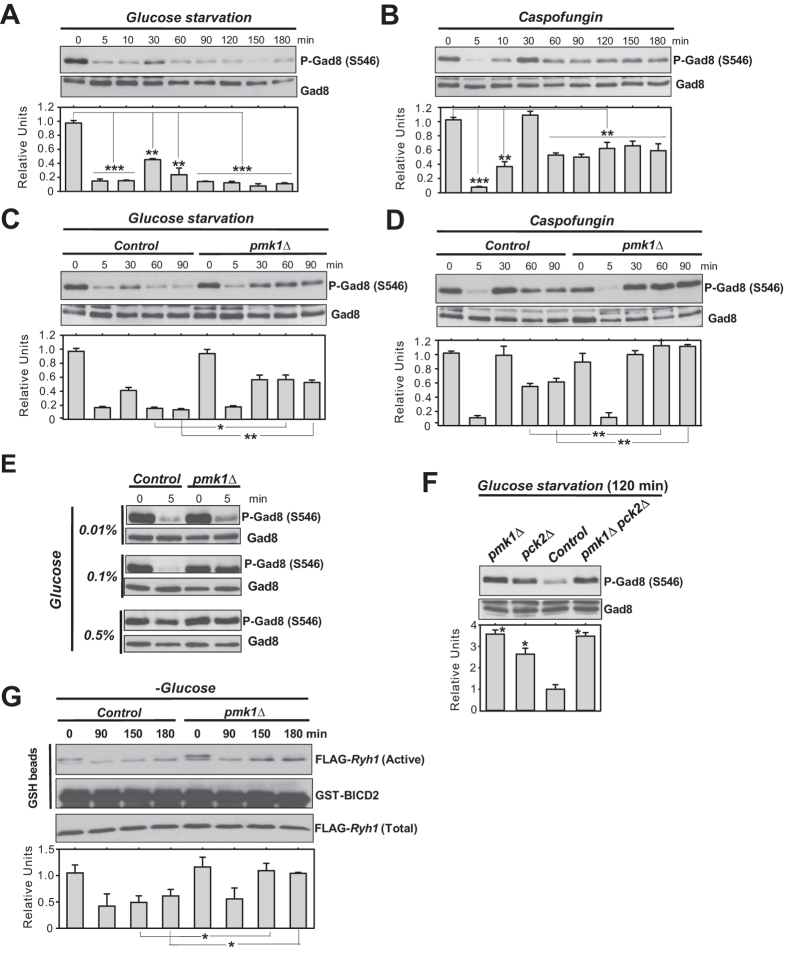
Pmk1 negatively modulates TORC2 signaling in response to stress by reducing Ryh1 activity. (**A**) Strain MM913 (control) was grown in YES medium and starved for glucose. Cell extracts were resolved by SDS-PAGE and S546-phosphorylated and total Gad8 detected with anti-phospho-S546 and anti-Gad8 antibodies, respectively. (**B**) Strain MM913 was grown in YES medium and treated with 1 μg/ml Caspofungin. S546-phosphorylated and total Gad8 were detected as above. (**C**) Strains MM913 (control) and MM1200 (*pmk1Δ*) were starved for glucose, and S546-phosphorylated and total Gad8 were detected as above. (**D**) Strains were grown in YES medium, treated with 1 μg/ml Caspofungin, and S546-phosphorylated and total Gad8 were detected as above. *P < 0.05; **P < 0.005; ***P < 0.001. (**E**) Strains were grown in YES medium and shifted to medium with 0.01%, 0.1%, or 0.5% glucose for 5 min. S546-phosphorylated and total Gad8 were detected as above. (**F**) Strains MI200 (control), MI102 (*pmk1Δ*), GB3 (*pck2Δ*), and BV544 (*pmk1Δ pck2Δ*) were grown in YES medium and shifted to the same medium lacking glucose for 120 min. S546-phosphorylated and total Gad8 were detected as above. *P < 0.05 in mutant strain as compared to control. (**G**) Strains CA6809 (control) and BV398 (*pmk1Δ*) expressing a genomic FLAG-Ryh1 fusion were grown in YES medium and starved for glucose. Bacterially purified GST-BICD2 was used for precipitation of active GTP-bound Ryh1 (active) in native non-denatured yeast extracts obtained at the indicated times. Precipitated Ryh1 and BICD2 (GSH beads) were detected with anti-FLAG and anti-GST antibodies, respectively. Total Ryh1 in cell extracts was detected with anti-FLAG antibody. *P < 0.05 in mutant strain as compared to control.

The results obtained so far indicate that Ryh1 positively regulates the activation of the CIP through TORC2-dependent and -independent mechanisms. Ryh1, which regulates TORC2-Gad8 activity in glucose-rich media, becomes inactive under glucose deprivation^22^. TORC2 activity, measured by using the *in vivo* phosphorylation status of Gad8 at serine 546 as readout, decreases rapidly in fission yeast cells starved for glucose, but recovers the initial levels after 30 minutes^22^,^30^. Congruent with these precedents, Gad8 phosphorylation at S546 decreased quickly in glucose-starved wild type cells (Fig. 6A). However, Gad8-S546 phosphorylation reached a maximum close to 50% of the initial level after 30 minutes in the absence of glucose, and this was followed by an additional decrease in phosphorylation that was maintained for longer incubation times (Fig. 6A). Notably, Gad8-S546 phosphorylation also decreased rapidly after 5 min of treatment with Caspofungin, regained the initial phosphorylation level after 30 minutes of incubation, and decreased again at longer times (Fig. 6B). Thus, in fission yeast the activation status of TORC2-Gad8 undergoes remarkable fluctuations that are dynamically similar, but quantitatively different depending on the type of stress. Importantly, Gad8-S546 phosphorylation changed similarly in control and *pmk1∆* cells during growth and after 30 minutes exposure to both stresses, but increased significantly in the *pmk1∆* mutant at longer incubation times (60–90 min; Fig. 6C,D). Moreover, contrary to control cells, the quick drop in Gad8-S546 phosphorylation was absent in *pmk1∆* cells when switched to osmotically equilibrated medium with 0.1% glucose (Fig. 6E). These findings suggest that Pmk1 down-regulates TORC2 signaling during the early and late responses to stress. Pck2 phosphorylates Gad8 *in vivo* to negatively regulate TORC2-Gad8 signaling^39^. Indeed, Gad8-S546 phosphorylation in the absence of glucose remained higher in *pck2∆* cells as compared to control cells, but was reproducibly lower than in glucose-starved *pmk1∆* and *pck2∆ pmk1∆* cells (Fig. 6F, Suppl. Figure S4). Thus, Pmk1 negatively regulates TORC2 activity by a mechanism that is independent of Pck2 and Gad8. The fact that *ryh1∆ pmk1∆* and *tor1∆ pmk1∆* double mutants are synthetic lethal, prevented from a direct examination of at which level Pmk1 is negatively regulating this signaling pathway. However, by performing pull-down assays as previously described^22^, we found that Ryh1 activity decreased during prolonged incubation times in control cells when switched to medium without glucose (Fig. 6G) and this correlated with the lower Gad8-S546 phosphorylation levels displayed at the same conditions (Fig. 6F). Remarkably, the decrease in GTPase activity was attenuated within the same time points in a *pmk1∆* background (Fig. 6F,G). Hence, Pmk1 negatively modulates TORC2 signaling in response to stress by reducing Ryh1 GTPase cycle.

### Phosphoinositide metabolism and Ryh1 crosstalk during control of TORC2 and CIP signaling

**Figure 7:**
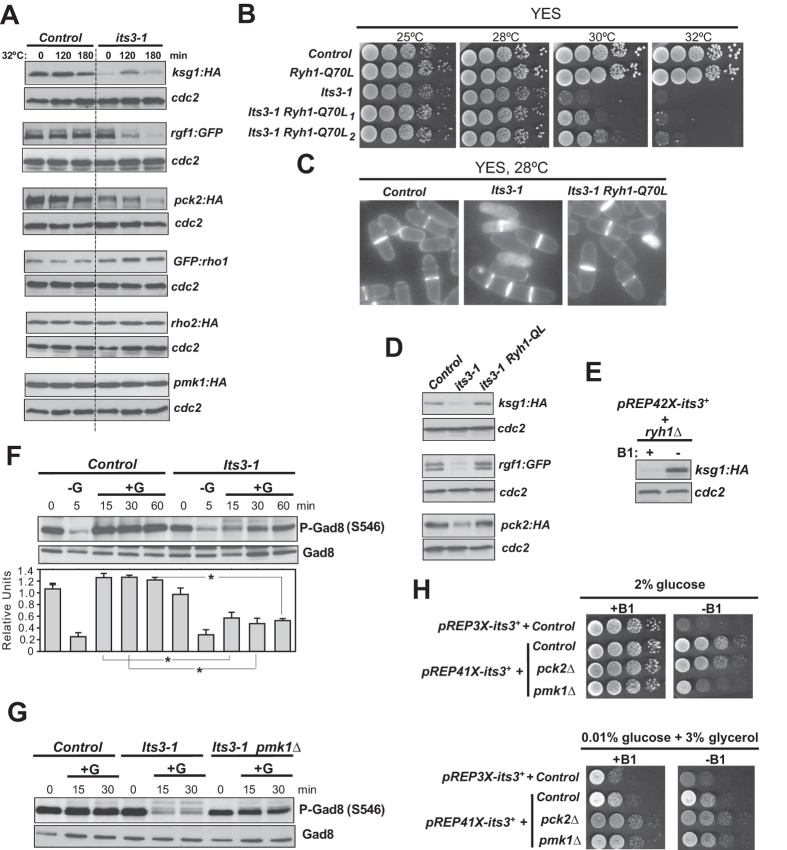
Its3 and Ryh1 crosstalk during control of TORC2 and CIP signaling. (**A**) Cultures of control and *its3-1* cells strains expressing Ksg1-HA, Rgf1-GFP, GFP-Rho1, Rho2-HA, Pck2-HA, or Pmk1-HA fusions were grown in YES medium at 25 °C and incubated at 32 °C (restrictive temperature) for the indicated times. Cell extracts were resolved by SDS-PAGE and protein levels of the respective fusions detected after incubation with anti-HA or anti-GFP antibodies. Anti-Cdc2 was used as loading control. (**B**) Serial dilutions of suspensions of strains MI200 (control), MM1300 (*its3-1*), CA6817 (Ryh1*-Q70L*) and BV574 (*its3-1* Ryh1*-Q70L*) were spotted on YES plates and incubated for 3 days at 25, 28, 30 and 32 °C. **(C)** Cultures of strains described in (B) were grown at 28 °C and observed by fluorescence microscopy after staining with calcofluor white. **(D)** Cultures of control, *its3-1* and *its3-1* Ryh1*-Q70L* cells expressing Ksg1-HA, Rgf1-GFP or Pck2-HA fusions were grown in YES medium at 25 °C and incubated at 32 °C for 2 hours. The respective fusions detected after incubation with anti-HA or anti-GFP antibodies. **(E)** Strain BV100 was transformed with pREP42X-*its3 *+ plasmid and grown in EMM2 medium at 28 °C in the presence or absence of thiamine (B1) for 24 h. Ksg1 and Cdc2 levels were detected as above. (**F**) Strains MI200 (control), and MM1300 (*its3-1*) were grown in YES medium at 25 °C, starved for glucose at 32 °C (−G), and then resuspended in YES medium with 0.2% glucose (+G) at 32 °C for the indicated times. S546-phosphorylated and total Gad8 were detected with anti-phospho-S546 and anti-Gad8 antibodies, respectively. (**G**) Strains MI200 (control), MM1300 (*its3-1*), and BV532 (*its3-1 pmk1Δ*) were grown in YES medium at 25 °C, starved for glucose at 32 °C (−G), and then resuspended in YES medium with 0.2% glucose (+G) at 32 °C for the indicated times. S546-phosphorylated and total Gad8 were detected as above. (**H**) Control and mutant strains were transformed separately with pREP3X-*its3 *+* *and pREP41X-*its3 *+* *plasmids, and serially diluted suspensions of the respective transformants were spotted on EMM2 + 2% glucose and EMM2 + 0.01% glucose + 3% glycerol plates with or without 5 μg/ml thiamine, and incubated for 3 (glucose plates) or 5 (glycerol plates) days at 28 °C.

Plasma membrane localization of Its3, which is an essential phosphatidylinositol-4-phosphate 5-kinase that synthesizes phosphatidylinositol (4,5)-bisphosphate (PI(4,5)P2) and regulates cell integrity, is reduced in *ryh1∆* cells^24^, but not in *tor1∆* or *pmk1∆* mutants (Suppl. Figure S5). Both Ksg1 and Rgf1 harbor phosphoinositide-binding pleckstrin homology (PH) domains at their C-termini, and Rgf1 localization at cell tips is diminished in cells expressing a thermosensitive *its3-1* allele^40^. Importantly, and similar to *ryh1∆* cells, Pmk1 activation induced by salt stress is also impaired in *its3-1* cells^41^. As shown in Fig. 7A, a temperature shift of growing *its3-1* cells from 25 to 32 °C (restrictive temperature) triggered a progressive decrease in Rgf1 levels, suggesting that Its3 function mediates Rgf1 localization and stability. Ksg1 levels were down-regulated in *its3-1* cells even at the permissive temperature, while Pck2 levels dropped in *its3-1* cells at longer incubation times at the restrictive temperature (Fig. 7A). Similar to *ryh1∆* cells, Rho1 levels were constitutively higher in *its3-1* cells as compared to control cells, whereas Rho2 and Pmk1 levels remained unchanged (Fig. 7A). Remarkably, simultaneous expression in *its3-1* cells of the GTP-locked Ryh1 allele Ryh1-*Q70L*^21^, or deletion of the Ryh1 GAP Gyp10^24^, partially suppressed the strong thermosensitive phenotype of *its3-1* cells (Fig. 7B; Suppl. Figure S6). The multiseptated and lytic phenotype of *its3-1* cells growing at the semipermissive temperature (28 °C) was clearly alleviated in *its3-1* Ryh1-*Q70L* cells (Fig. 7C). In addition, Ksg1, Rgf1 and Pck2 protein levels were restored in *its3-1* Ryh1-*Q70L* cells as compared to *its3-1* cells (Fig. 7D), and in *ryh1∆* cells after moderated Its3 expression (42X thiamine-repressible promoter; Fig. 7E). Therefore, Ryh1 may regulate processing and/or plasma membrane trafficking of some upstream regulatory members of the CIP through a mechanism involving Its3 and PI(4,5)P2.

PI4P and PI(4,5)P2 metabolism control TORC2 signaling in budding yeast^42^. Compared to control cells, Gad8-S546 phosphorylation recovery in glucose starved cells transferred to glucose-rich medium was defective in *its3-1* cells (Fig. 7F), supporting that proper PI(4,5)P2 turnover regulates TORC2 signaling in fission yeast. Remarkably, Gad8-S546 phosphorylation recovery in glucose-rich medium was enhanced in a *its3-1 pmk1∆* double mutant with respect to *its3-1* cells (Fig. 7G). Hence, Its3 may separately promote CIP and TORC2-Gad8 signaling in response to environmental cues.

Strong Its3 overexpression (3X thiamine-repressible promoter) is lethal in fission yeast^24^. Contrariwise, moderated Its3 expression under the 41X promoter version (~60X lower fold expression level than the 3X version) was not lethal in wild type or *pck2∆* cells, but promoted an evident growth defect in absence of Pmk1 (Fig. 7H). However, this effect was not observed when Its3-overexpressing *pmk1∆* cells were grown in a non-fermentable carbon source like glycerol, where Ryh1 activity is low (Fig. 7H). A straight confirmation that deregulated Ryh1 GTPase cycle provokes the growth defect of *pmk1∆* cells in these conditions was not possible since a double deletion of Pmk1 and Ryh1 is synthetic lethal (data not shown). However, the above results indicate that Pmk1-mediated downregulation of Ryh1 is biologically relevant in the presence of glucose, when its activity is high.

## Discussion

Increased *de novo* synthesis of the PKC ortholog Pck2 promotes downstream signaling and increased activation of MAPK Pmk1, the core member of the CIP, in response cell wall damage or absence of glucose^27^,^29^. The TORC2 kinase Tor1 participates in this mechanism, as demonstrated by the strong defect in Pck2 synthesis and Pmk1 activation displayed by *tor1Δ* cells during growth and in response to both stimuli^29^. We now extend this previous observation to show that Tor1 control of the CIP is partially executed through its main target, the Akt ortholog Gad8. This somehow unexpected finding is supported by the observation that Pck2 levels and Pmk1 activation became partially abrogated in *gad8Δ* cells subjected to cell wall damage or glucose exhaustion. However, defective Pck2 levels were less evident in *gad8Δ* cells than in the *tor1Δ* mutant, implying that TORC2 may also activate the CIP by additional Gad8-independent mechanisms. Tor-dependent phosphorylation at turn motif promotes stabilization of some conventional and novel mammalian PKC isoforms^43^. However, *in vivo* turn motif phosphorylation of Pck2 at T984 occurs by an autophosphorylation mechanism, and Pck2 protein levels are very similar in cells expressing wild type or a Pck2-*T984A* allele^29^. Therefore, the lower defective Pck2 levels displayed by *gad8Δ* cells do not seem to result from Tor1-dependent phosphorylation and stabilization of the kinase. Gad8 phosphorylates Tor1 at threonine-1972 within the ATP-binding domain to reduce its activity, and Tor1-*T1972A* cells display increased activity and stress resistance^44^. Enhanced Tor1 activity in absence of negative feedback loop regulation by Gad8 might account for the increased Pck2 levels observed in stressed *gad8Δ* cells as compared to the *tor1Δ* mutant. Interestingly, Pmk1 activation during cell wall damage or glucose starvation was similar in both *tor1Δ* and *gad8Δ* mutants despite the fact that Pkc2 levels are lower in the Tor1-less mutant. Low Pck2 levels and MAPK activation are still detected in cycloheximide–treated cells or in *tor1∆* cells subjected to both stresses^27^ (this work). Moreover, Pmk1 activation does not rely exclusively on Pck2-dependent signaling to the MAPK module, and other known (Rho1; Pck1) and unknown players are involved in this response^27^,^28^. It could be possible that the expected differences in MAPK activation between *tor1∆* and *gad8∆* cells might be too low to be detected in the context of our experimental setup due to a remnant MAPK activation occurring independently of Tor1-Gad8 control of Pck2 levels. Alternatively, the presence of cellular Pck2 levels at a certain range (like those in *tor1∆* or *gad8∆* cells) might be sufficient to promote identical MAPK activation under both stimuli. mTORC2 is physically associated with actively translating ribosomes^32^,^45^. We also observed that both Tor1 and Gad8 associate *in vivo* to translating ribosomes in fission yeast. Hence, although the possibility that non-ribosomal TORC2 and Gad8 may upregulate Pck2 synthesis in response to stress cannot be ruled out, our observations strongly suggest that this process might be executed by ribosome-based TORC2 and Gad8. Moreover, we found that the S6K ortholog and TORC1-target Psk1 also associates with translating ribosomes and collaborates with Tor1 and Gad8 in the control of Pck2 levels, Pmk1 activation, and cellular adaptation to stress. However, this role is clearly less prominent than that of the TORC2-Gad8 branch, and only emerges when TORC1 and *psk1Δ* mutants combine with a *gad8 *^+^* *deletion. mTORC1 regulates protein synthesis by controlling translational initiation and preserving eukaryotic translation initiation factor 2-alpha (eIF2α) in a dephosphorylated state^6^. Interestingly, inhibition of TORC1 by nutrient deprivation induces phosphorylation of eIF2α in fission yeast^15^. Psk1 might thus reinforce Pck2 synthesis and stress response by keeping low eIF2α phosphorylation levels.

In higher eukaryotes Rps6 phosphorylation changes upon a wide variety of stimuli to fine-tune efficient translation of mRNAs and protein synthesis^34^. However, a fission yeast mutant expressing a non phosphorylated version of Rps6 was insensitive to stress, and showed no defects in Pck2 levels and Pmk1 activation under stress. Thus, Rps6 phosphorylation mediated by TORC1 (Psk1) and TORC2 (Gad8) is not biologically relevant during the stress response in this organism. Recent global polysome and ribosome profiling studies in budding yeast have also failed to uncover a role of Rps6 phosphorylation in global or individual mRNAs translation^46^.

**Figure 8:**
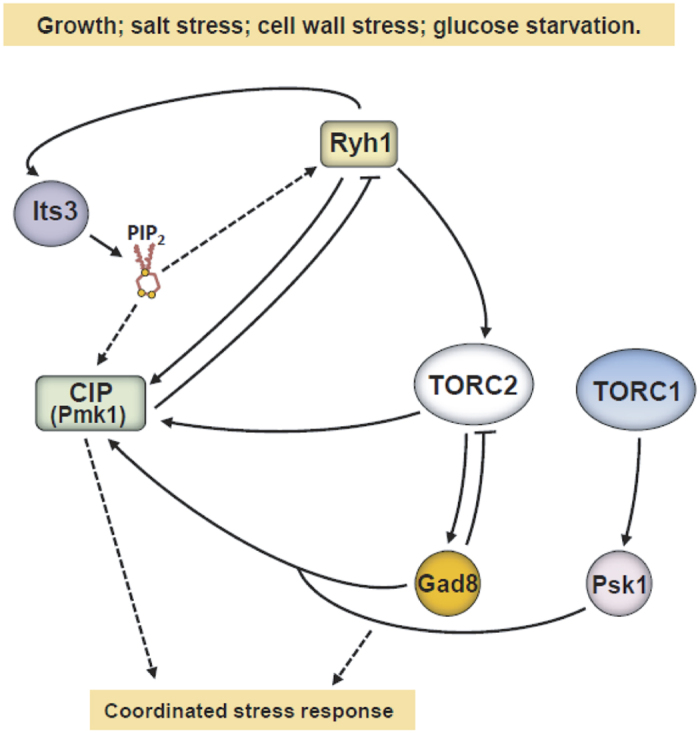
Cross-regulatory interactions between TOR and cell integrity MAPK signaling in fission yeast. Rab GTPase Ryh1 cross-activates the cell integrity pathway (CIP) through two independent mechanisms. TORC2 target Gad8 cooperates with TORC1 target Psk1 to increase Pck2 protein levels and promote activation of MAPK Pmk1during growth and stress. Alternatively, Ryh1 elicits plasma membrane targeting and/or stabilization of several upstream activators of the CIP (Ksg1 (PDK), Rgf1 (Rho1 GEF), and Pck2). PI kinase Its3 and PI(4,5)P2 promote Ryh1-TORC2 signaling and activation of the CIP, and in this later case might act as Ryh1 effector. In addition, activated Pmk1 decreases TORC2-Gad8 signaling in response to stress by downregulating Ryh1 activation cycle. Coordinated activation/deactivation of TOR and CIP signaling allows precise cell adaptation to multiple environmental cues.

Ryh1 regulates the intra-Golgi trafficking and is involved in recycling from endosome to the Golgi, as well as in trafficking events from the Golgi to the plasma membrane^23^,^25^,^47^. Here we obtained strong evidence supporting that Ryh1 controls the activity of the CIP through an additional mechanism independent of its role as an upstream regulator of TORC2 signaling (Fig. 8). This is based in two main findings. First, contrary to control, *tor1Δ*, or *gad8Δ psk1Δ* cells, Pmk1 activation in response to a salt stress was strongly defective in *ryh1Δ* cells. Second, protein levels and/or plasma membrane localization of key upstream regulatory members of the CIP, including PDK ortholog Ksg1, Rho1 GEF Rgf1, Rho GTPases Rho1 and Rho2, and Pck2, are heavily misregulated in *ryh1Δ* cells with respect to control, *tor1Δ*, and *gad8Δ* cells. Consequently, Ryh1 function might be required for proper trafficking of the above signaling proteins to their membrane anchors. Intriguingly, membrane targeting and protein levels of Ksg1, Rho1, and Rho2 were also moderately reduced in *tor1Δ* and *gad8Δ* cells, suggesting that TORC2-Gad8 might regulate to some extent plasma membrane trafficking and/or protein stabilization events. In support of this possibility, the plasma membrane localization of the high affinity glucose transporter Ght5 under low-glucose conditions is regulated by TORC2-Gad8^48^. Collectively, our results reveal that Ryh1 positively controls the activation of the CIP via two distinct branches (Fig. 8). One involves proper trafficking and localization of key activators of the CIP, and is particularly relevant during MAPK activation in response to osmotic stress. The second branch is essential during cell wall damage or absence of glucose, and reinforces MAPK activation via TORC2-Gad8 to enhance Pck2 levels. In any case, this novel role of Ryh1 as elicitor of MAPK signaling might not be shared by budding yeast, since deletion of Ypt6 (Ryh1 ortholog) did not impair activation of MAPKs Slt2 and Hog1 in response to different stimuli (Suppl. Figure S7).

We found that PI4P 5-kinase Its3 is essential for TORC2-Gad8 activation in the presence of glucose, and this function is independent of its role as a positive regulator of the CIP. Conversely, adequate plasma membrane trafficking of Its3 requires Ryh1 function^24^, and similar to *ryh1∆* cells, Ksg1, Rgf1, Rho1, and Pck2 levels were strongly impaired in cells with defective Its3 function. Remarkably, the thermosensitive phenotype, morphological defects and low Ksg1, Rgf1 and Pck2 levels of *Its3-1* cells were partially restored in the presence of a constitutive Ryh1 activity. Moreover, increased Its3 expression prompted a clear recovery in Ksg1 levels in *ryh1∆* cells. Therefore, besides positively regulating Ryh1-TORC2 signaling, Its3 and PI(4,5)P2 might operate downstream Ryh1 during regulation of trafficking and localization of activators of the CIP (Fig. 8). In budding yeast, PI kinases may regulate PDK orthologs Pkh1/2 and TORC2 at the plasma membrane^42^. Hence, this dual functional role of PI kinases might be evolutionary conserved.

Similar to glucose starvation^22^,^30^, TORC2-Gad8 becomes quickly deactivated and reactivated during cell wall stress; however, in both instances this recovery is followed by a progressive decrease in activity. Moreover, Pmk1 is a critical positive regulator of these dynamic oscillations in TORC2-Gad8 signaling, as they become attenuated in the absence of the MAPK. Importantly, our results strongly suggest that Pmk1 exerts this control upstream of Ryh1 by downregulating its GTPase cycle. Sat1 (Rgp1) and Sat4 (Ric1) form a complex that acts as GEF for Ryh1^21^, whereas Gyp10 is a GAP for the GTPase^24^. As compared to *gyp10∆* cells, Gad8-S546 phosphorylation remained higher after prolonged incubation in the absence of glucose in a double *pmk1∆ gyp10∆* mutant (Suppl. Figure S8), implying that Gyp10 is not targeted by Pmk1 during regulation of Ryh1 activity. Expression levels and phosphorylation status of Sat1-myc and Sat4-myc genomic fusions remained unchanged in control versus *pmk1∆* cells in response to glucose deprivation (Suppl. Figure S8). However, both proteins harbor several putative MAPK phosphor sites that might become phosphorylated *in vivo* by Pmk1 to inhibit GEF activity towards Ryh1. Although the exact nature of Pmk1 mediated cross-inhibition of Ryh1 function is currently unknown, it appears biologically relevant because negative regulation of Ryh1-TORC2 by the CIP ensures cell survival in the presence of glucose when Its3 function becomes upregulated under moderate Its3 overexpression. Activation of TORC2 by active GTP-bound Ryh1 may take place at the plasma membrane^21^. In budding yeast several CIP components participate in the control of plasma membrane fluidity homeostasis^49^. Thus, the CIP might act in fission yeast as a mediator of plasma membrane integrity and/or homeostasis to optimize Ryh1-TORC2-Gad8 signaling during growth and stress. Simultaneous deletion of the CIP and TORC2 components induces a synthetic sick or lethal phenotype in fission yeast^29^, pointing that they share one or more functions during growth. Our findings draw a scenario where the activity of both pathways is coordinated in a highly sophisticated fashion through multiple cross-activation and cross-inhibition events to allow precise cell adaptation to multiple environmental cues (Fig. 8; a more detailed version of this model is shown in Suppl. Figure S9).

## Methods

### Strains, gene disruption, growth conditions, and stress treatments

Yeast strains used in this work are shown in S1 Table. The *S. pombe tor1*^+^, *gad8*^+^, *psk1*^+^, *gyp10*^+^ and *ryh1*^+^ null mutants were obtained by ORF deletion and replacement with the *hphMX6* (hphR), the G418 (*kanR*) or the *NatMX6* (NatR) cassettes by PCR-mediated strategy using plasmids pFA6a-*hphMX6*, pFA6a-*kanMX6*, or pFA6a-*NatMX6*^50^. Strains expressing different genomic fusions in multiple genetic backgrounds were constructed either by transformation or after random spore analysis of appropriate crosses. Yeast strains were grown in rich (YES) or minimal (EMM2) medium with 2% glucose plus supplements^51^. Strains expressing fusions under the control of the medium (41X) or strong (3X) thiamine inducible promoter (*nmt1*) were grown in liquid EMM2 with thiamine (5 mg/L), and transferred to the same medium lacking thiamine for 18–24 hours. In osmotic-saline and cell-wall stress experiments log-phase cultures (OD_600_ = 0.5) were supplemented with either KCl (Sigma-Aldrich) or Caspofungin (Sigma-Aldrich). In glucose starvation experiments cells grown in YES medium with 7% glucose were recovered by filtration, and resuspended in the same medium lacking glucose and osmotically equilibrated with 3% glycerol, or with 0.01, 0.1 or 0.5% glucose osmotically equilibrated with glycerol, depending on the particular experiment. In TOR inhibition experiments, Torin (Cell Signaling Technology) was added to cultures to a final concentration of 50 μM and incubated for one hour. At different times the cells from 50 ml of culture were harvested by centrifugation at 4 °C, washed with cold PBS buffer, and the yeast pellets immediately frozen in liquid nitrogen for further analysis.

*S. cerevisiae* strains were grown in YPD medium at 30 °C to early log-phase (OD_600_ = 0.5), and incubated at 39 °C (heat stress), or treated with either 0.5 M NaCl (Sigma-Aldrich), 12 mM Caffeine (Sigma-Aldrich), 10 μg/ml Calcofluor White (Sigma-Aldrich) or 1 μg/ml Caspofungin. Cells from 50 ml of culture were collected at different times and processed as above.

### Detection of total Pck2

Cell extracts were prepared under native conditions using Buffer IP (50 mM Tris-HCl (pH 7.5), 5 mM EDTA, 150 mM NaCl, 1 mM β- mercaptoethanol, 10% glycerol, 0.1 mM sodium orthovanadate, 1% Triton X-100, and protease inhibitors). Equal amounts of total protein were resolved in 6% SDS-PAGE gels and transferred to Hybond-ECL membranes. Total Pck2 was detected with mouse monoclonal anti-HA antibody (Roche Molecular Biochemicals). Mouse monoclonal anti-PSTAIR (anti-Cdc2, Sigma-Aldrich) was used for loading control. Immunoreactive bands were revealed with an anti-mouse-HRP-conjugated secondary antibody (Sigma-Aldrich) and the ECL system (GE-Healthcare).

### Detection of Tor1, Rho1, Rho2, Rgf1, Cpc2, Psk1, Sat1, Sat4 and Ksg1-tagged fusions

Cells extracts were prepared using Buffer IP as above and resolved in 8, 10, or 12% SDS-PAGE gels depending on the size of the fused protein. HA-Tor1 and Rho2-HA were detected with mouse monoclonal anti-HA antibody (12CA5). Rabbit polyclonal anti-GFP (Cell Signaling) was employed to detect Rgf1-GFP, Rho1-GFP and Cpc2-GFP fusions. Sat1-13myc, Sat4-13myc and Psk1-13myc fusions were detected with a mouse monoclonal anti-c-myc antibody (clone 9E10, Roche Molecular Biochemicals). Immunoreactive bands were revealed with anti-rabbit or anti-mouse-HRP-conjugated secondary antibodies (Sigma-Aldrich) and the ECL system (GE-Healthcare). A mouse monoclonal HRP-conjugated anti-HA antibody (Sigma-Aldrich) was used for detection of the Ksg1-HA fusion.

### Purification and detection of activated Pmk1 and Sty1

Preparation of cell extracts, affinity chromatography purification of HA-tagged Pmk1 or Sty1 with Ni^2+^ -NTA-agarose beads (Qiagen), and SDS-PAGE was performed as described^26^. This approach alleviates the potential inaccuracy in the detection of both total and phosphorylated MAPKs. Dual phosphorylation in either Pmk1 or Sty1 was detected employing rabbit polyclonal anti-phospho-p44/42 (Cell Signaling) or mouse monoclonal anti-phospho-p38 (Cell Signaling), respectively. Total Pmk1 or Sty1 were detected with mouse monoclonal anti-HA antibody. Immunoreactive bands were revealed with anti-rabbit or anti-mouse-HRP-conjugated secondary antibodies (Sigma-Aldrich) and the ECL system (GE-Healthcare).

### Detection of activated Slt2 and Hog1 in *S. cerevisiae*

Total cell extracts were obtained as described in ref. 52, and dual phosphorylation in either Slt2 or Hog1 was detected employing, respectively, anti-phospho-p44/42 or anti-phospho-p38 antibodies as described above. Mouse monoclonal anti-PSTAIR (anti-Cdc2, Sigma-Aldrich) was used for loading control.

### Detection of total and S546-phosphorylated Gad8

Cells were fixed and total protein extracts prepared by precipitation with trichloroacetic acid (TCA) as previously described^53^. Proteins were resolved in 10% SDS-PAGE gels and transferred to Hybond-ECL membranes. An anti-phospho-polyclonal antibody produced by immunization of rabbits with a synthetic phospho-peptide corresponding to residues surrounding Ser546 of Gad8 (GenScript) was used to detect TORC2-dependent phosphorylation of Gad8 at S546. Total Gad8 was detected after incubation with a rabbit polyclonal antibody obtained after immunization with a peptide corresponding to N-terminal end of Gad8 (GenScript). Immunoreactive bands were revealed with anti-rabbit HRP-conjugated secondary antibody (Sigma) and the ECL system (GE-Healthcare).

### Ryh1 GTPase pull-down assays

Determination of Ryh1 activity was performed essentially as described in ref. 22. A GST-fused version of a 706–814 amino-acid region of human Bicaudal D2 (BICD2) was obtained after cloning the corresponding DNA fragment (GenScript) into plasmid pGEX-KG to generate pGEX-KG-BICD2. The GST-BICD2 fusion was expressed in *E. coli* and purified by affinity chromatography with gluthatione Sepharose beads (GE-Healthcare). Fission yeast cells expressing N-terminal FLAG-tagged Ryh1 were lysed in ice-cold PBS containing 10 mM MgCl_2_, 0.5% Tween 20, 1 mM PMSF, and the protease inhibitor cocktail for use in purification of histidine-tagged proteins (Sigma-Aldrich). Crude cell lysate was cleared and the resulting supernatant incubated for 45 minutes with GST-BICD2 immobilized on glutathione beads. Following incubation, the beads were washed 3 times with PBS containing 10 mM MgCl_2_ and 0.5% Tween 20. Either total or BICD2-bound Ryh1 was resolved in 12% SDS-PAGE gels, and detected after incubation with a rat monoclonal anti-FLAG antibody (Sigma-Aldrich) followed by immunodetection with anti-rat HRP-conjugated secondary antibody (Sigma) and the ECL system (GE-Healthcare).

### Quantification of Western blot experiments and reproducibility of results

Densitometric quantification of Western blot signals as of 16-bit.jpg digital images of blots was performed using ImageJ^54^. Briefly, bands plus background were selected or drawn as rectangles and a profile plot was obtained for each band (peaks). To minimize the background noise in the bands, each peak floating above the baseline of the corresponding profile plot was manually closed off using the straight-line tool. This tool was also employed to adjust the closing at the base of the peak in the particular case of the spill-over signals. Finally, measurement of the closed peaks was performed with the wand tool. Experiments were repeated at least three times with similar results. Mean relative units+SD and/or representative results are shown.

### Expression of human Rab6A in *S. pombe*

cDNA encoding human Rab6A was amplified by PCR using a plasmid PM100 as template (a gift from Prof. Bruno Goud, Institute Curie, France) and the oligonucleotide HARab6-F (ATGTATCCCTATGACGTCCCGGACTATGCAATGTCCACGGG CGGAGACTT), which hybridizes at the start of *rab6*^+^ ORF and incorporates a 27-nucleotide sequence (underlined) encoding one HA epitope (sequence YPYDVPDYA), and Rab6-EcoR1-R (TATAT*GAATTC*TTAGC AGGAACAGCCTCCTT), which hybridizes at the 3′ end of *rab6*^+^ ORF and incorporates a *EcoR*I site. Ryh1 promoter was amplified by PCR using fission yeast genomic DNA as template and 5′-oligonucleotide ProRyh1-BamHI-F (TATAT*GGATCC*TGCGTAGATT ATGAAACATTCTAATAAAAT), which hybridizes at positions 795 to 765 upstream of the *ryh1*^+^ ATG start codon and contains a *BamH*I site, and 3′-oligonucleotide ProRyh1HA-R (TGCATAGTCCGGGACGTCATAGGGATACATTAT GACAAAAAGTTTTGACTTCTAAATATA), which hybridizes at the end of Ryh1 promoter and incorporates a 27-nucleotide sequence (underlined) encoding one HA epitope. Rab6 and Ryh1 promoter PCR fragments were used as template for a third PCR using 5′-oligonucleotide ProRyh1-BamHI-F and 3′-oligonucleotide Rab6-EcoR1-R. The resulting PCR fragments were purified, digested with *BamH*I and *EcoR*I, and cloned into the integrative plasmid PJK210. The resulting plasmid was digested at the unique *Stu1* site within *ura4*^+^, and transformed into *ryh1Δ* strain BV385 (Table S1). Transformants expressing *ura4*^+^ were obtained and the fusions verified by both PCR and Western blot analysis.

### Preparation and fractionation of polysomes

Strains exponentially growing in YES medium or EMM2 medium without thiamine were treated with 100 μg/ml of cycloheximide prior to centrifugation. The pellets were washed, resuspended in lysis buffer (10 mM Tris-HCl, pH 7.5, 100 mM NaCl, 30 mM MgCl_2_, 100 μg/ml cycloheximide) and supplemented with a protease inhibitor cocktail (Sigma) and 0.5 U/μl of RNAse inhibitor (RNasin, Promega). Total cell homogenates were obtained in a Fast-Prep instrument (Bio 101) with chilled acid-washed glass beads, and the crude extracts clarified by centrifugation at 20000 × *g* for 30 min. 20 OD_260_ units of clarified extracts were applied to 11.2 ml of 7–47% (w/v) sucrose gradients in lysis buffer plus inhibitors and centrifuged in a SW41Ti rotor (Beckman instruments) at 4 °C for 3.5 hours at 40000 rpm. Fractions of 600 μl were collected and 40 μl aliquots used to detect the appropriated protein fusions by SDS-PAGE and Western blot analysis.

### Plate assay of stress sensitivity for growth

Decimal dilutions of control and mutant strains were spotted per duplicate on usual YES solid medium or supplemented with different concentrations of MgCl_2_ (Sigma-Aldrich), KCl (Sigma-Aldrich), glucose, or Caspofungin (Sigma-Aldrich). Plates were incubated at 28 °C for 3 days (5 days in low-glucose plates) and then scanned.

### Fluorescence microscopy

Images of cells expressing GFP-Rho1, GFP-Rho2, Rgf1-GFP, and GFP-CRB fusions were taken on a Leica DM 4000B fluorescence microscope with a 100x objective and captured with a cooled Leica DC 300 F camera and IM50 software. To measure fluorescence distribution at plasma membrane or cell tips, the percentage of GFP intensity at the cell periphery/tips with respect to the total GFP intensity in the cell was calculated using ImageJ. Briefly, for each cell (N > 15), ROI (region of interest) were drawn with the freehand selection tool to encompass the whole cell and the cytoplasm. Once corrected for background fluorescence, ROI area was measured, and fluorescence at plasma membrane/poles was then calculated by subtracting the cytoplasm value from the whole cell value, and expressed as percentage per cell.

## Additional Information

**How to cite this article**: Madrid, M. *et al*. Multiple crosstalk between TOR and the cell integrity MAPK signaling pathway in fission yeast. *Sci. Rep.* **6**, 37515; doi: 10.1038/srep37515 (2016).

**Publisher's note:** Springer Nature remains neutral with regard to jurisdictional claims in published maps and institutional affiliations.

## Supplementary Material

Supplementary Information
