# Supplementary material for: Multiple crosstalk between TOR and the cell integrity MAPK signaling pathway in fission yeast
Source: Sci Rep. 2016 Nov 23;6:37515. doi: 10.1038/srep37515 (PMC5120329; doi:10.1038/srep37515)
Supplement: Supplementary Information [file srep37515-s1.pdf]

Supplemental material for:

Multiple crosstalk between TOR and the cell integrity MAPK signaling pathway in fission yeast.

Marisa Madrid[1*], Beatriz Vázquez-Marín[1], Alejandro Franco, Teresa Soto, Jero Vicente-Soler, Mariano Gacto, and José Cansado[*].

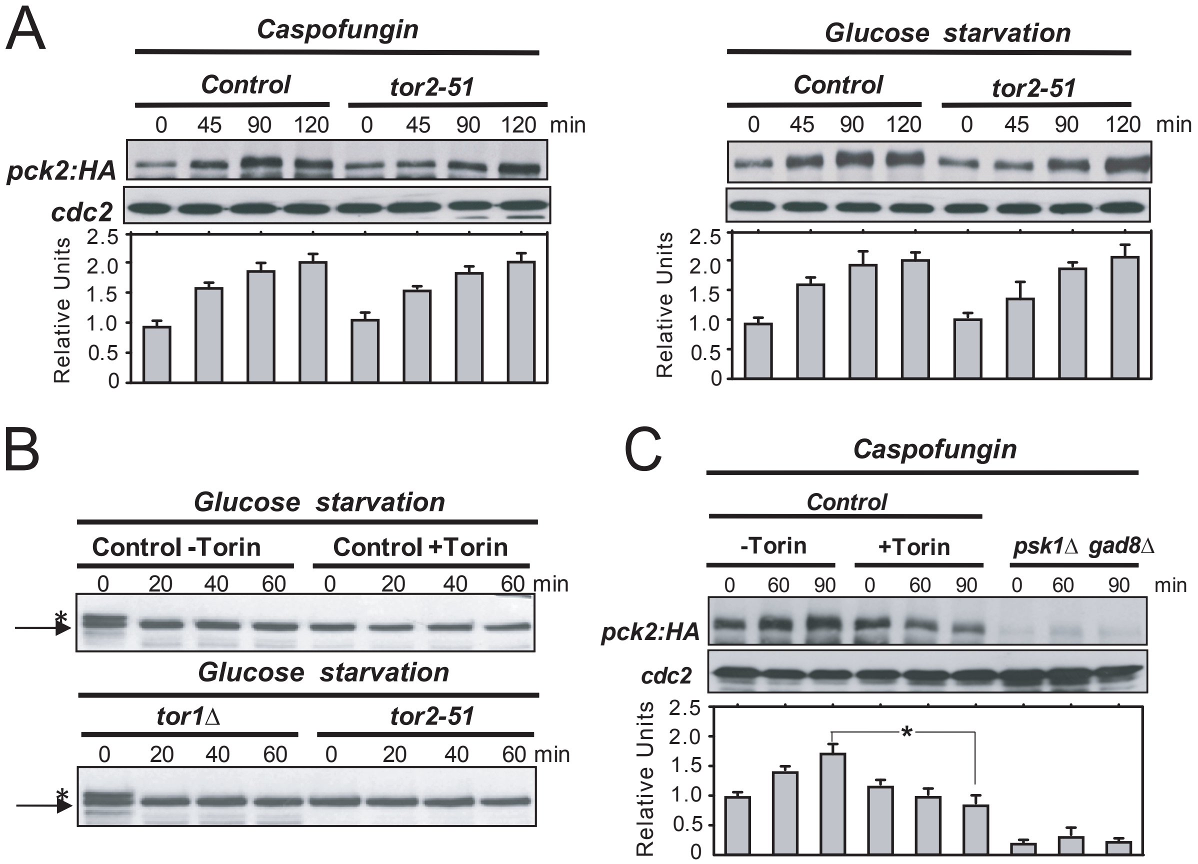

Supplementary Figure S1: (**A**) Growing cultures of strains BV8 (Pck2-HA; control) and BV60 (*tor2-51*, Pck2-HA) were grown in YES medium and treated with 1µg/ml Caspofungin, or shifted to the same medium without glucose and supplemented with 3% glycerol. Cell extracts were resolved by SDS-PAGE and Pck2 levels were detected after incubation with anti-HA antibodies. Anti-Cdc2 was used as a loading control.

(**B**) Strains AN0179 (Psk1-13myc; control), BV91 (*tor1*Δ Psk1-13myc), and BV92 (*tor2-51* Psk1-13myc), were grown in YES medium, pre-incubated with or without 50 µM Torin for 60 min (control cells), or at 36ºC for one hour (*tor2-51* cells), and shifted to the same medium without glucose and supplemented with 3% glycerol. The Psk1-13myc fusion was detected after incubation with anti-myc antibodies. *Arrow*, unphosphorylated Psk1; *asterisk*, TORC1-phosphorylated Psk1.

(**C**) A growing culture of control strain BV8 (Pck2-HA) was first incubated with 50 µM Torin for 60 min or remained untreated (DMSO). These cells, together with a culture of strain BV14 (*psk1*Δ *gad8*Δ Pck2-HA) were treated afterwards with 1 µg/ml Caspofungin for the indicated times. Pck2 was detected as described above. *,$P<0.05$ in Torin–treated cells as compared to the untreated culture.

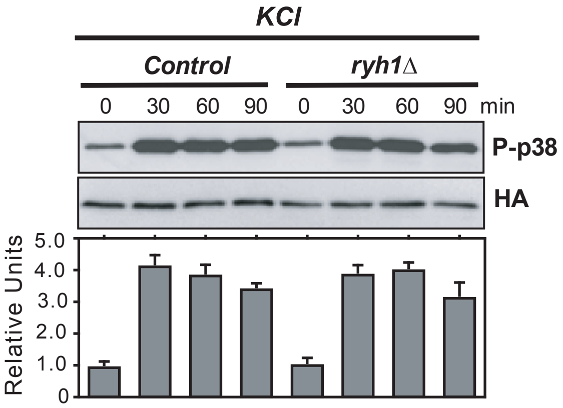

Supplementary Figure S2: Growing cultures of strains JM1250 (control) and BV133 (*ryh1*Δ) expressing genomic Sty1-HA6H fusions were treated with 0.6 M KCl. Sty1 fusion was purified by affinity chromatography, and activated/total Sty1 was detected with anti-phospho-p38 and anti-HA antibodies, respectively.

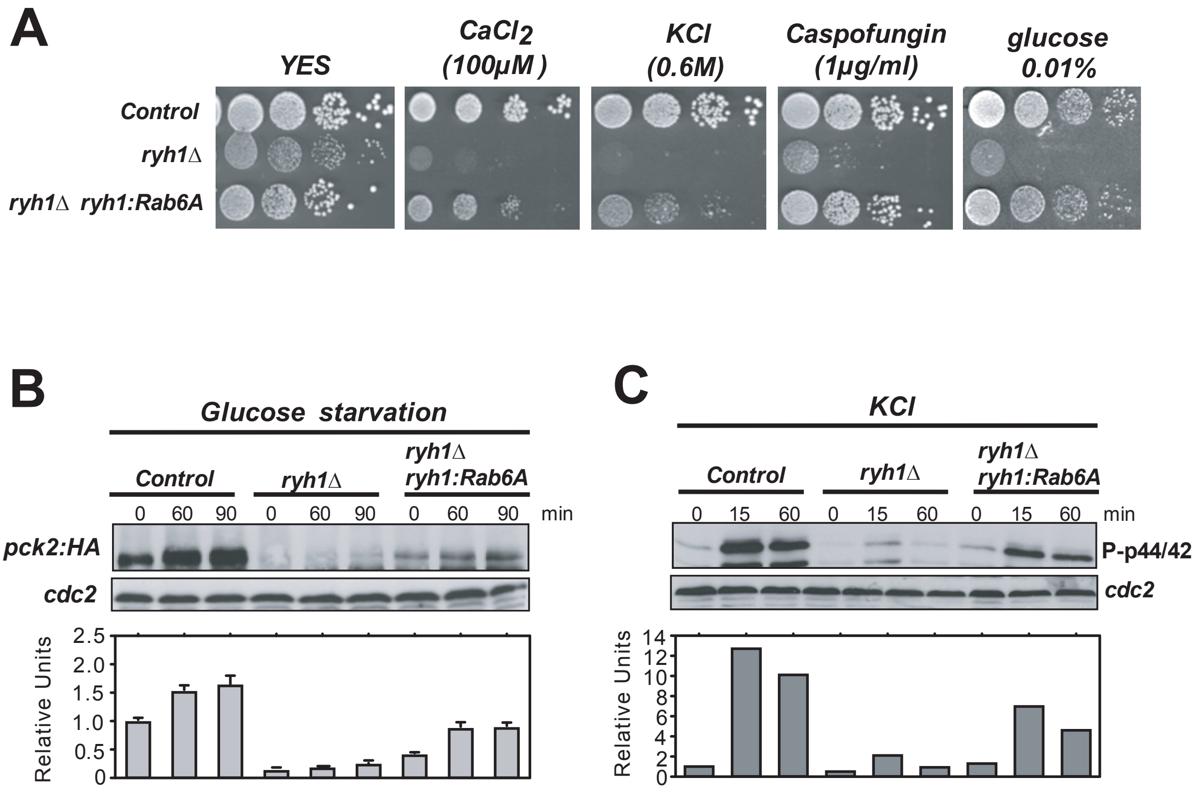

Supplementary Figure S3: (**A**) Serial dilutions of suspensions of strains BV8 (Pck2-HA; control), BV385 (*ryh1*Δ Pck2-HA), and BV403 (*ryh1*Δ, Rab6A-HA Pck2-HA), were spotted on YES plates supplemented with different concentrations of either $CaCl_2$, KCl, Caspofungin, or low glucose, and incubated for 3 or 5 days (low glucose plates) at 28ºC. (**B**) Growing cultures of strains described in (**A**) were grown in YES medium with 7% glucose and then shifted to the same medium without glucose and supplemented with 3% glycerol. Cell extracts were resolved by SDS-PAGE and Pck2 levels were detected after incubation with anti-HA antibodies. Anti-Cdc2 was used as a loading control. (**C**) Growing cultures of strains described in (**A**) were treated with 0.6 M KCl. Activated Pmk1 was detected with anti-phospho-p44/42 antibody, whereas anti-Cdc2 was used as a loading control.

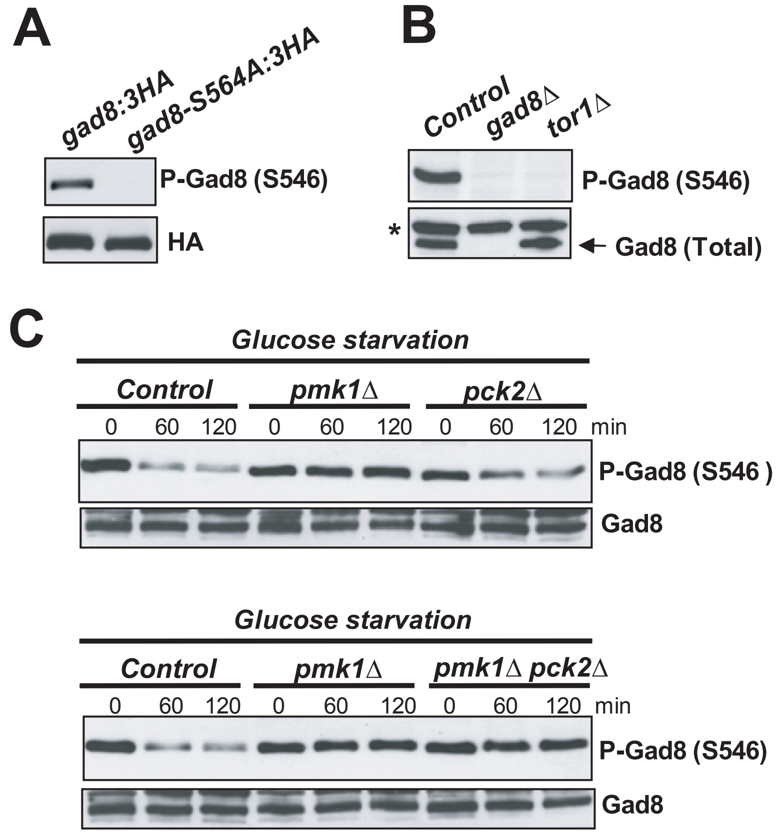

Supplementary Figure S4: (**A**) Strains JW960 (Gad8-3HA) and JW963 (Gad8-S546A-3HA) were grown to early-log phase in YES medium at 28ºC. S546-phosphorylayed and total Gad8 were detected with anti-phospho-S546 and anti-HA antibodies, respectively. (**B**) Strains MM913 (Control), BV11 (*gad8*Δ) and MM1205 (*tor1*Δ) were grown to early-log phase, and phosphorylayed and total (arrow) Gad8 were detected with anti-phospho S546 and anti-Gad8 antibodies, respectively. Asterisk indicates a nonspecific protein band. (**C**) Strains MI200 (control), MI102 (*pmk1*Δ), GB3 (*pck2*Δ), and BV544 (*pmk1*Δ *pck2*Δ) were grown in YES medium with 7% glucose and shifted to the same medium lacking glucose for the indicated times. S546-phosphorylated and total Gad8 were detected with anti-phospho-S546 and anti-Gad8 antibodies, respectively.

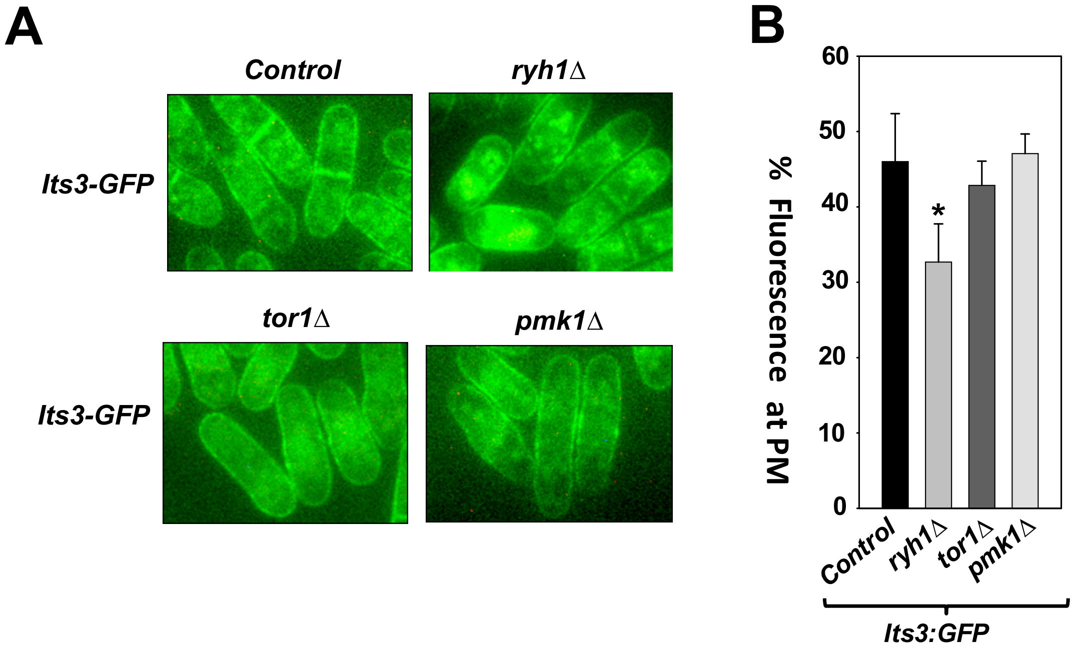

Supplementary Figure S5: Images by fluorescence microscopy of growing control, *ryh1*Δ*, tor1*Δ, and *pmk1*Δ cells expressing a genomic Its3-GFP fusion. The percentage at the plasma membrane of the GFP fusion with respect to total cell fluorescence was determined. N>15 cells; *, *P*<0.05

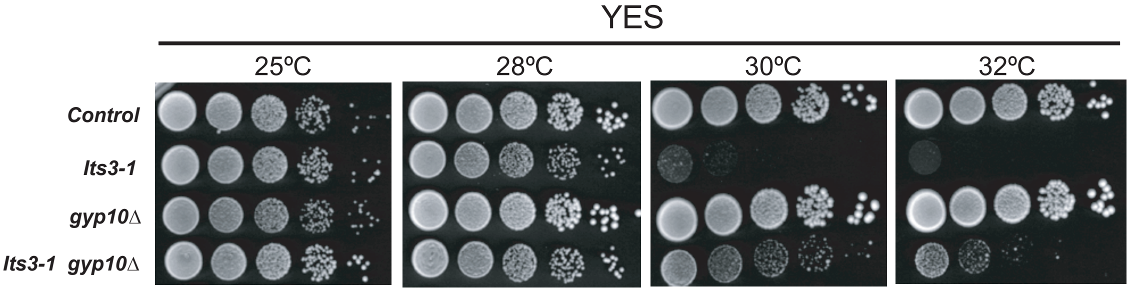

Supplementary Figure S6: Serial dilutions of suspensions of strains MI200 (control), MM1300 (*its3-1*), BV430 (*gyp10*Δ), and BV698 (*its3-1 gyp10*Δ), were spotted on YES plates and incubated for 3 days at 25, 28, 30 and 32ºC.

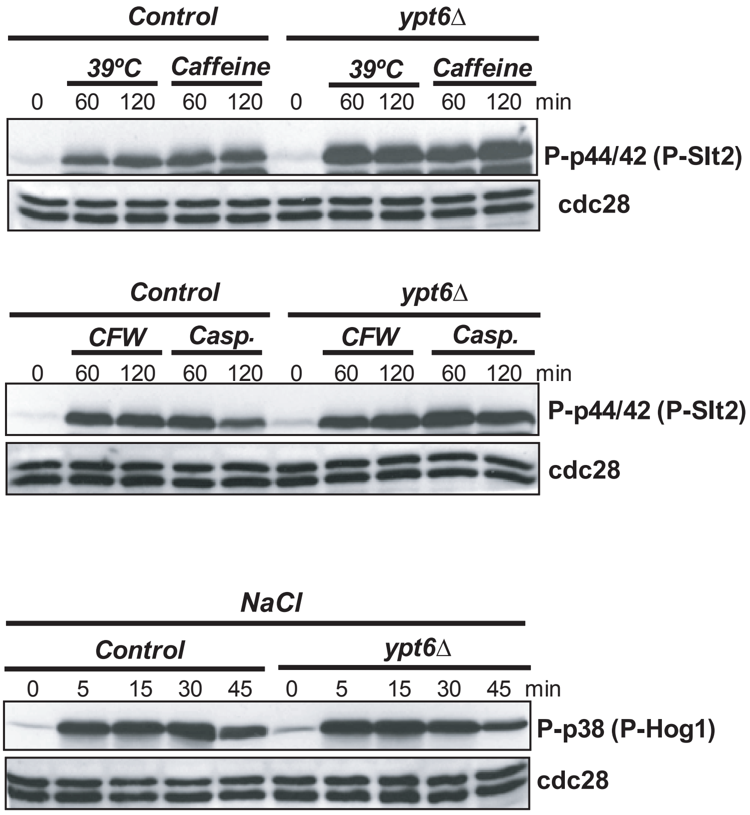

Supplementary Figure S7: Growing cultures of *S. cerevisiae* strains BY4741 (Control) and Y05171 (*ypt6*Δ) were incubated at 39ºC, or treated with either 12 mM Caffeine, 10 μg/ml Calcofluor White (CFW), 1 μg/ml Caspofungin (Casp.) or 0.5 M NaCl. Activated Slt2 and Hog1 were detected with anti-phospho-p44/42 and anti-phospho-p38 antibodies, respectively, whereas anti-Cdc2 (Cdc28) was used as a loading control.

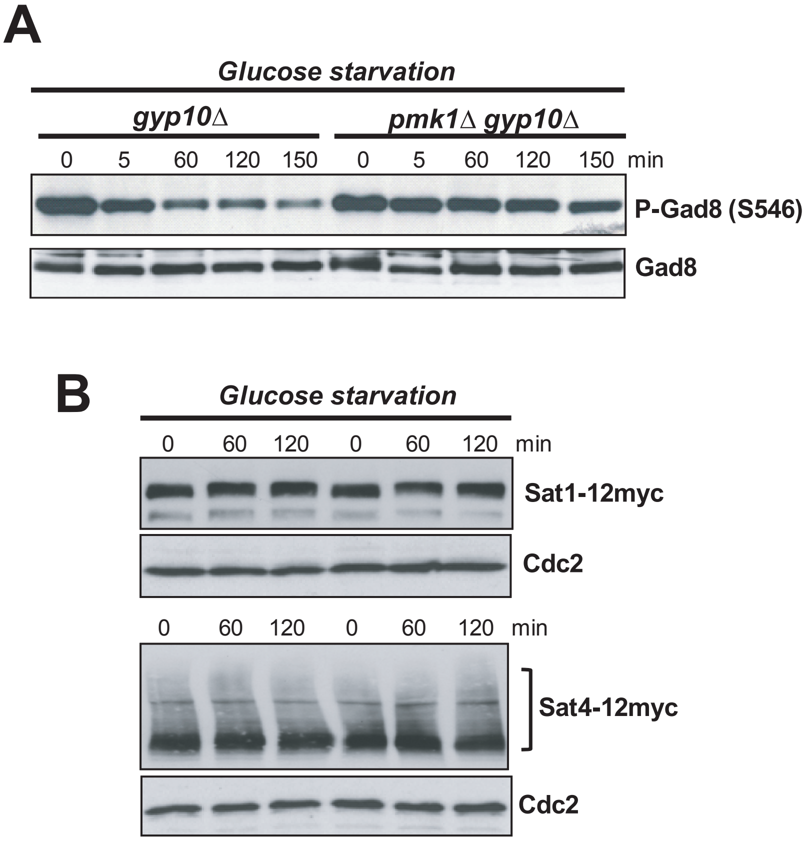

Supplementary Figure S8: (**A**) Strains BV430 (*gyp10*Δ) and BV482 (*pmk1*Δ *gyp10*Δ) were grown in YES medium with 7% glucose and shifted to the same medium lacking glucose for the indicated times. S546-phosphorylated and total Gad8 were detected with anti-phospho-S546 and anti-Gad8 antibodies, respectively. (**B**) Strains BV365 (Sat1-12myc) and BV367 (Sat4-12myc) were grown in YES medium with 7% glucose and shifted to the same medium lacking glucose for the indicated times. Total Sat1 and Sat4 levels were detected with anti-myc antibodies. Anti-Cdc2 was used as a loading control.

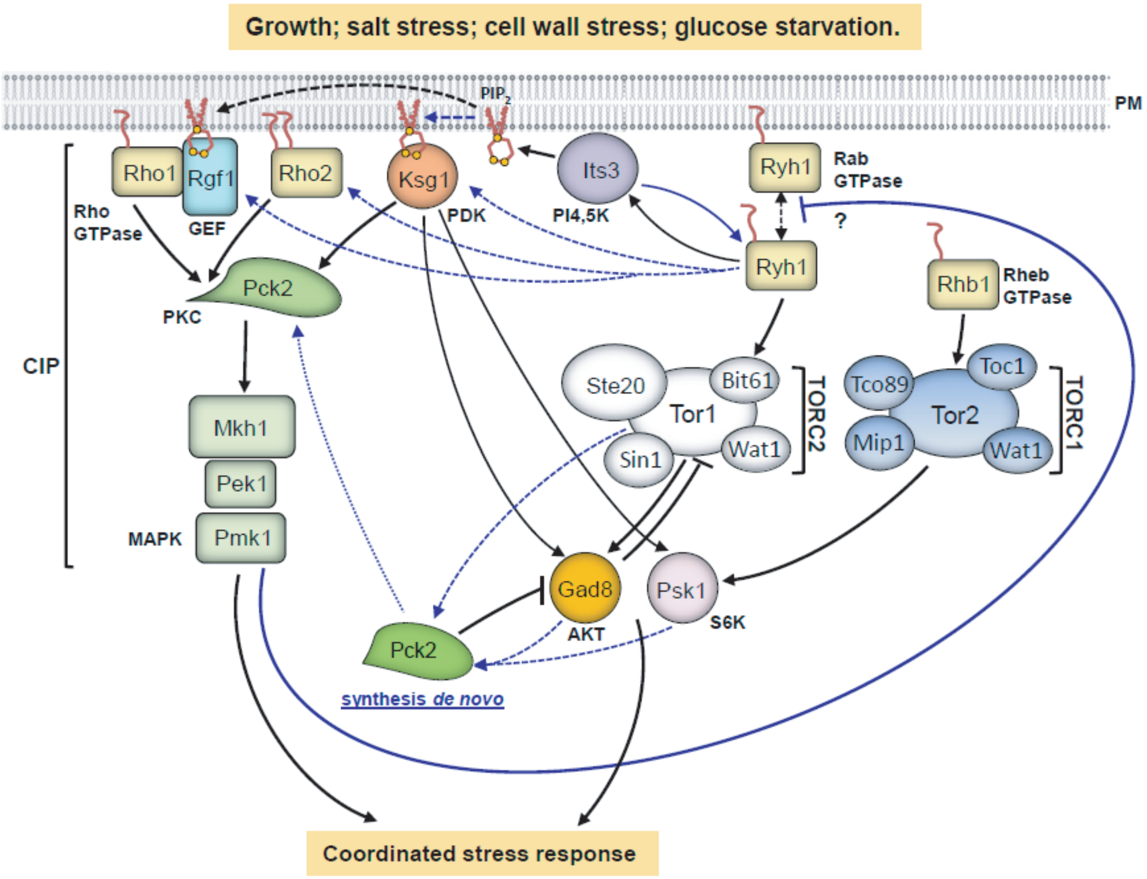

Supplementary Figure S9: Expanded model showing the cross-regulatory interactions between TOR and cell integrity MAPK signaling in fission yeast. For specific details please see text. Blue lines indicate novel functional interactions described in this work.

Supplementary Table S1: Strains used in this work.

***S.pombe* strains**[a]	**Genotype**	**Source/Reference**
MM913	h^{+} *pck2::KanR pck2-HA:leu1*$^{+}$ *pmk1-HA6H:ura4*$^{+}$	Madrid *et al.* (2015)
BV8	h^{-} *pck2::natR pck2-HA:leu1*$^{+}$ *pmk1-HA6H:ura4*$^{+}$	This work
MM1205	h^{+} *tor1::hphR pck2::kanR pck2-HA:leu1*$^{+}$ *pmk1-HA6H:ura4*$^{+}$	Madrid *et al.* (2015)
BV11	h^{-} *gad8:: natR pck2::KanR pck2-HA:leu1*$^{+}$ *pmk1-HA6H: ura4*$^{+}$	This work
BA120	h^{+} *tor2-51:ura4*$^{+}$	Álvarez and Moreno (2006)
BV60	$h^{?}$ *tor2-51 pck2::natR pck2-HA:leu1*$^{+}$	This work
BV369	$h^{?}$ *tor2-51 gad8::hygR pck2::natR pck2-HA:leu1*$^{+}$ *pmk1-HA6H:ura4*$^{+}$	This work
BV13	h^{-} *psk1::kanR pck2::natR pck2-HA:leu1*$^{+}$ *pmk1-HA6H:ura4*$^{+}$	This work
BV14	$h^{?}$*gad8::hygR psk1::kanR pck2::natR pck2-HA:leu1*$^{+}$ *pmk1-HA6H:ura4*$^{+}$	This work
AN0228	h^{-} *rps601::hygR rps602-S235S236AA:kanR*	Nakashima *et al.* (2010)
BV78	$h^{?}$ *rps601::hygR rps602-S235S236AA:kanR pck2::natR pck2-HA:leu1*$^{+}$ *pmk1-HA6H:ura4*$^{+}$	This work
AN0179	h^{-} *psk1-13myc:hphR*	Nakashima *et al.* (2012)
CA6817	h^{-} *3FLAG:ryh1Q70L*	Tatebe *et al.* (2010)
BV90	h^{-} *psk1-13myc:hphR 3FLAG:ryh1Q70L*	This work
BV91	h^{-} *psk1-13myc:hphR tor1::hygR*	This work
BV92	h^{-} *psk1-13myc:hphR tor2-51:ura4*$^{+}$	This work
BA192	h^{+} *kanR:nmt1:HA-tor1*	Álvarez and Moreno (2006)
JW960	h^{90} *gad8-6HA:kanR gad8::ura4*$^{+}$	National BioResource Project, Japan
JW963	h^{90} *gad8.S546A-6HA:kanR gad8::ura4*$^{+}$	National BioResource Project, Japan
AN071	h^{+} *cpc2::kanR cpc2-GFP:leu1*$^{+}$ *pmk1-HA6H:ura4*$^{+}$	Núñez *et al.* (2009)
BV397	$h^{?}$ *tor1::hygR gad8-6HA:kanR gad8::ura4*$^{+}$	This work
BV84	$h^{?}$ *tor1::natR*$^{-}$ *psk1-13myc:hphR*	This work
MI102	h^{+} *pmk1::kanR*	Madrid *et al.* (2006)
BV38	$h^{?}$ *ryh1::hygR pck2::natR pck2-HA:leu1*$^{+}$ *pmk1-HA6H:ura4*$^{+}$	This work
BV56	h^{+} *3FLAG:ryh1Q70L pck2::KanR pck2-HA:leu1*$^{+}$ *pmk1-HA6H:ura4*$^{+}$	This work
JM1250	h^{-} *sty1-HA6H:ura4*$^{+}$	Jonathan Millar
BV133	h^{-} *ryh1::hygR sty1-HA6H:ura4*$^{+}$	This work
BV94	h^{+} *ksg1-HA:leu1*$^{+}$	This work
BV100	$h^{?}$ *ryh1::hygR ksg1-HA:leu1*$^{+}$	This work
BV245	$h^{?}$ *tor1::hygR ksg1-HA:leu1*$^{+}$	This work
BV354	$h^{?}$ *gad8::hygR ksg1-HA:leu1*$^{+}$	This work
PPG118.20	h^{+} *rgf1-GFP:leu1*$^{+}$ *rgf1::his2*$^{+}$	Pilar Pérez
BV341	$h^{?}$ *ryh1::hygR rgf1-GFP:leu1*$^{+}$ *rgf1::his2*	This work
BV339	$h^{?}$ *tor1::hygR rgf1-GFP:leu1*$^{+}$ *rgf1::his2*	This work
BV350	$h^{?}$ *gad8::hygR rgf1-GFP:leu1*$^{+}$ *rgf1::his2*	This work
BV110	h^{+} *rho1-GFP:leu1*$^{+}$	Pilar Pérez
BV193	$h^{?}$ *ryh1::hygR rho1-GFP:leu1*$^{+}$ *pmk1-HA6H:ura4*$^{+}$	This work
BV247	$h^{?}$ *tor1::natR rho1-GFP:leu1*$^{+}$ *pmk1-HA6H:ura4*$^{+}$	This work
BV248	$h^{?}$ *gad8::hygR rho1-GFP:leu1*$^{+}$ *pmk1-HA6H:ura4*$^{+}$	This work
LSM400	h^{+} *rho2-HA:leu1*$^{+}$ *rho2::kanR*	Sánchez-Mir *et al.* (2014)
BV95	h^{+} *ryh1::hygR rho2-HA:leu1*$^{+}$ *rho2::kanR*	This work
BV243	h^{+} *tor1::hygR rho2-HA:leu1*$^{+}$ *rho2::kanR*	This work
BV352	h^{+} *gad8::hygR rho2-HA:leu1*$^{+}$ *rho2::kanR*	This work
BV524	h^{+} *pmk1-HA6H:ura4*$^{+}$ *pREP41X-Ksg1-GFP*	This work

BV526	*h*[+] *tor1::natR pmk1-HA6H:ura4*[+] *pREP41X-Ksg1-GFP*	This work
BV525	*h*[+] *ryh1::hygR pmk1-HA6H:ura4*[+] *pREP41X-Ksg1-GFP*	This work
LSM300	*h*[+] *rho2-GFP:leu1*[+] *rho2::kanR pmk1-HA6H: ura4*[+]	Sánchez-Mir *et al.* (2014)
BV249	*h*[+] *tor1::natR rho2-GFP:leu1*[+] *rho2::kanR pmk1-HA6H: ura4*[+]	This work
BV195	*h*[+] *ryh1::hygR rho2-GFP:leu1*[+] *rho2::kanR pmk1-HA6H: ura4*[+]	This work
CA5931	*h*[-] *CRIB-GFP:ura4*[+]	Tatebe *et al.* (2008)
BV271	*h*[-] *tor1::hygR CRIB-GFP:ura4*[+]	This work
BV241	*h*[-] *ryh1::hygR CRIB-GFP:ura4*[+]	This work
MM1273	*h*[-] *its3-1*	James Moseley
BV574	*h*[-] *its3-1 3FLAG:ryh1Q70L*	This work
BV498	*h*[?] *its3-1 ksg1-HA:leu1*[+]	This work
MM2151	*h*[?] *its3-1 ksg1-HA:leu1*[+] *3FLAG:ryh1Q70L*	This work
BV484	*h*[?] *its3-1 rgf1-GFP:leu1*[+] *rgf1::his2*	This work
MM2159	*h*[?] *its3-1 rgf1-GFP:leu1*[+] *3FLAG:ryh1Q70L*	This work
MM1836	*h*[?] *its3-1 pck2::KanR pck2-HA:leu1*[+] *pmk1-HA6H:ura4*[+]	This work
MM2166	*h*[?] *its3-1 pck2::KanR pck2-HA:leu1*[+] *3FLAG:ryh1Q70L pmk1-HA6H:ura4*[+]	This work
BV493	*h*[?] *its3-1 rho1-GFP:leu1*[+]	This work
BV489	*h*[?] *its3-1 rho2-HA:leu1*[+] *rho2::kanR*	This work
MM1200	*h*[+] *pmk1::natR pck2::KanR pck2-HA:leu1*[+] *pmk1-HA6H:ura4*[+]	This work
MI200	*h*[+] *pmk1-HA6H:ura4*[+]	Madrid *et al.* (2006)
GB3	*h*[+] *pck2::kanR pmk1-HA6H:ura4*[+]	Barba *et al.* (2008)
BV544	*h*[?] *pmk1::natR pck2::kanR*	This work
CA6809	*h*[-] *3FLAG:ryh1*	Hatano *et al.* (2015)
BV398	*h*[?] *pmk1::kanR 3FLAG:ryh1*	This work
BV168	*h*[?] *pmk1::kanR 3FLAG:ryh1Q70L*	This work
MM1300	*h*[?] *pmk1-HA6H::ura4 its3-1*	Kabeche *et al.* (2015)
BV532	*h*[?] *its3-1 pmk1::kanR*	This work
BV385	*h*[-] *ryh1::hygR pck2::kanR pck2-HA:leu1*[+] *ura4.294*	This work
BV403	*h*[-] *ryh1::hygR pck2::kanR pck2-HA:leu1*[+] *rab6-HA:ura4*[+] *ura4.294*	This work
BV404	*h*[-] *ryh1::hygR pck2::kanR pck2-HA:leu1*[+] *rab6-HA:ura4*[+] *ura4.294*	This work
BV430	*h*[+] *gyp10::kanR*	This work
BV698	*h*[-] *its3-1 gyp10::kanR*	This work
BV482	*h*[+] *gyp10::kanR pmk1::natR*	This work
BV365	*h*[+] *sat1-12myc:kanR*	This work
BV367	*h*[+] *sat4-12myc:kanR*	This work
***S.cerevisiae* strains**	**Genotype**	**Source/Reference**
BY4741	*MATa his3Δ1 leu2Δ0 met15Δ0 ura3Δ0*	EUROSCARF
Y05171	*MATa his3Δ1 leu2Δ0 met15Δ0 ura3Δ0 ypt6::kanMX4*	EUROSCARF

[a] All strains are *ade- ura4D-18 leu1-32*
